# A Genome-Wide Screen for Promoter Methylation in Lung Cancer Identifies Novel Methylation Markers for Multiple Malignancies 

**DOI:** 10.1371/journal.pmed.0030486

**Published:** 2006-12-26

**Authors:** David S Shames, Luc Girard, Boning Gao, Mitsuo Sato, Cheryl M Lewis, Narayan Shivapurkar, Aixiang Jiang, Charles M Perou, Young H Kim, Jonathan R Pollack, Kwun M Fong, Chi-Leung Lam, Maria Wong, Yu Shyr, Rita Nanda, Olufunmilayo I Olopade, William Gerald, David M Euhus, Jerry W Shay, Adi F Gazdar, John D Minna

**Affiliations:** 1 Hamon Center for Therapeutic Oncology Research, The University of Texas Southwestern Medical Center at Dallas, Dallas, Texas, United States of America; 2 Simmons Comprehensive Cancer Center, The University of Texas Southwestern Medical Center at Dallas, Dallas, Texas, United States of America; 3 Department of Pharmacology, The University of Texas Southwestern Medical Center at Dallas, Dallas, Texas, United States of America; 4 Department of Surgery, The University of Texas Southwestern Medical Center at Dallas, Dallas, Texas, United States of America; 5 Department of Pathology, The University of Texas Southwestern Medical Center at Dallas, Dallas, Texas, United States of America; 6 Department of Biostatistics, Vanderbilt University School of Medicine, Nashville, Tennessee, United States of America; 7 Department of Genetics, Lineberger Comprehensive Cancer Center, University of North Carolina at Chapel Hill, Chapel Hill, North Carolina, United States of America; 8 Department of Pathology and Laboratory Medicine, University of North Carolina at Chapel Hill, Chapel Hill, North Carolina, United States of America; 9 Department of Pathology, Stanford University, Stanford, California, United States of America; 10 Department of Thoracic Medicine, The Prince Charles Hospital, University of Queensland, Brisbane, Australia; 11 University Department of Medicine, Queen Mary Hospital, The University of Hong Kong, Hong Kong Special Administrative Region, China; 12 Section of Hematology/Oncology, Department of Medicine, University of Chicago, Chicago, Illinois, United States of America; 13 Department of Pathology, Memorial Sloan-Kettering Cancer Center, New York, New York, United States of America; 14 Department of Cell Biology, The University of Texas Southwestern Medical Center at Dallas, Dallas, Texas, United States of America; 15 Department of Internal Medicine, The University of Texas Southwestern Medical Center at Dallas, Dallas, Texas, United States of America; University of California San Francisco, United States of America

## Abstract

**Background:**

Promoter hypermethylation coupled with loss of heterozygosity at the same locus results in loss of gene function in many tumor cells. The “rules” governing which genes are methylated during the pathogenesis of individual cancers, how specific methylation profiles are initially established, or what determines tumor type-specific methylation are unknown. However, DNA methylation markers that are highly specific and sensitive for common tumors would be useful for the early detection of cancer, and those required for the malignant phenotype would identify pathways important as therapeutic targets.

**Methods and Findings:**

In an effort to identify new cancer-specific methylation markers, we employed a high-throughput global expression profiling approach in lung cancer cells. We identified 132 genes that have 5′ CpG islands, are induced from undetectable levels by 5-aza-2′-deoxycytidine in multiple non-small cell lung cancer cell lines, and are expressed in immortalized human bronchial epithelial cells. As expected, these genes were also expressed in normal lung, but often not in companion primary lung cancers. Methylation analysis of a subset (45/132) of these promoter regions in primary lung cancer (*n* = 20) and adjacent nonmalignant tissue (*n* = 20) showed that 31 genes had acquired methylation in the tumors, but did not show methylation in normal lung or peripheral blood cells. We studied the eight most frequently and specifically methylated genes from our lung cancer dataset in breast cancer (*n* = 37), colon cancer (*n* = 24), and prostate cancer (*n* = 24) along with counterpart nonmalignant tissues. We found that seven loci were frequently methylated in both breast and lung cancers, with four showing extensive methylation in all four epithelial tumors.

**Conclusions:**

By using a systematic biological screen we identified multiple genes that are methylated with high penetrance in primary lung, breast, colon, and prostate cancers. The cross-tumor methylation pattern we observed for these novel markers suggests that we have identified a partial promoter hypermethylation signature for these common malignancies. These data suggest that while tumors in different tissues vary substantially with respect to gene expression, there may be commonalities in their promoter methylation profiles that represent targets for early detection screening or therapeutic intervention.

## Abstract 2

John Minna and colleagues report that a group of genes are commonly methylated in primary lung, breast, colon, and prostate cancer.

## Editors' Summary

**Background.:**

Tumors or cancers contain cells that have lost many of the control mechanisms that normally regulate their behavior. Unlike normal cells, which only divide to repair damaged tissues, cancer cells divide uncontrollably. They also gain the ability to move round the body and start metastases in secondary locations. These changes in behavior result from alterations in their genetic material. For example, mutations (permanent changes in the sequence of nucleotides in the cell's DNA) in genes known as oncogenes stimulate cells to divide constantly. Mutations in another group of genes—tumor suppressor genes—disable their ability to restrain cell growth. Key tumor suppressor genes are often completely lost in cancer cells. But not all the genetic changes in cancer cells are mutations. Some are “epigenetic” changes—chemical modifications of genes that affect the amount of protein made from them. In cancer cells, methyl groups are often added to CG-rich regions—this is called hypermethylation. These “CpG islands” lie near gene promoters—sequences that control the transcription of DNA into RNA, the template for protein production—and their methylation switches off the promoter. Methylation of the promoter of one copy of a tumor suppressor gene, which often coincides with the loss of the other copy of the gene, is thought to be involved in cancer development.

**Why Was This Study Done?:**

The rules that govern which genes are hypermethylated during the development of different cancer types are not known, but it would be useful to identify any DNA methylation events that occur regularly in common cancers for two reasons. First, specific DNA methylation markers might be useful for the early detection of cancer. Second, identifying these epigenetic changes might reveal cellular pathways that are changed during cancer development and so identify new therapeutic targets. In this study, the researchers have used a systematic biological screen to identify genes that are methylated in many lung, breast, colon, and prostate cancers—all cancers that form in “epithelial” tissues.

**What Did the Researchers Do and Find?:**

The researchers used microarray expression profiling to examine gene expression patterns in several lung cancer and normal lung cell lines. In this technique, labeled RNA molecules isolated from cells are applied to a “chip” carrying an array of gene fragments. Here, they stick to the fragment that represents the gene from which they were made, which allows the genes that the cells express to be catalogued. By comparing the expression profiles of lung cancer cells and normal lung cells before and after treatment with a chemical that inhibits DNA methylation, the researchers identified genes that were methylated in the cancer cells—that is, genes that were expressed in normal cells but not in cancer cells unless methylation was inhibited. 132 of these genes contained CpG islands. The researchers examined the promoters of 45 of these genes in lung cancer cells taken straight from patients and found that 31 of the promoters were methylated in tumor tissues but not in adjacent normal tissues. Finally, the researchers looked at promoter methylation of the eight genes most frequently and specifically methylated in the lung cancer samples in breast, colon, and prostate cancers. Seven of the genes were frequently methylated in both lung and breast cancers; four were extensively methylated in all the tumor types.

**What Do These Findings Mean?:**

These results identify several new genes that are often methylated in four types of epithelial tumor. The observation that these genes are methylated in multiple independent tumors strongly suggests, but does not prove, that loss of expression of the proteins that they encode helps to convert normal cells into cancer cells. The frequency and diverse patterning of promoter methylation in different tumor types also indicates that methylation is not a random event, although what controls the patterns of methylation is not yet known. The identification of these genes is a step toward building a promoter hypermethylation profile for the early detection of human cancer. Furthermore, although tumors in different tissues vary greatly with respect to gene expression patterns, the similarities seen in this study in promoter methylation profiles might help to identify new therapeutic targets common to several cancer types.

**Additional Information.:**

Please access these Web sites via the online version of this summary at http://dx.doi.org/10.1371/journal.pmed.0030486.

- US National Cancer Institute, information for patients on understanding cancer
- CancerQuest, information provided by Emory University about how cancer develops
- Cancer Research UK, information for patients on cancer biology
- Wikipedia pages on epigenetics (note that Wikipedia is a free online encyclopedia that anyone can edit)
- The Epigenome Network of Excellence, background information and latest news about epigenetics

## Introduction

Tumor-acquired alterations in DNA methylation include both genome-wide hypomethylation and locus-specific hypermethylation. Genomic hypomethylation occurs early in cellular transformation and affects both genome stability and imprinted gene expression [1–3]. Promoter hypermethylation often coincides with loss of heterozygosity at the same locus, which can result in the loss of function of the gene in tumor cells. These genetic and epigenetic changes often occur at tumor suppressor gene loci, and are hypothesized to participate in cancer development [4].

While genomic methylation patterns are clearly deranged in cancer cells, the DNA methyltransferases themselves are rarely if ever mutated or aberrantly expressed [5]. The “rules” governing which genes are methylated during the pathogenesis of individual cancers, as well as the timing of their methylation and silencing (e.g., during preneoplasia or in metastatic progression) are unknown, and it is not yet clear how specific methylation patterns are initially established in tumor cells [6,7]. However, aberrant promoter hypermethylation is common to most tumors, and in many cases, appears to have tumor-type specificity [8]. A few genes, such as the cyclin-dependent kinase inhibitor *(p16)* and the tumor suppressor gene *ras association domain family protein 1A (RASSF1A)* are methylated across many tumor types, but they appear to be exceptions. Identification of more genes of this type would represent a common promoter hypermethylation profile for multiple carcinomas [9–12].

In the present study, we employ gene expression profiling of lung cancer cells and immortalized human bronchial epithelial cells (HBECs) and contrast their expression phenotype before and after 5-aza-2′-deoxycytidine (5-aza) treatment to identify genes subject to frequent promoter hypermethylation in human cancers. Since CpG island methylation is readily detectable in tissues and fluids, the identification of a promoter hypermethylation gene set that is common to multiple malignancies—with high frequency and specificity for tumors compared to normal tissues—would have important implications for patient screening, diagnosis, and therapeutic intervention [12,13].

## Methods

### Cell Lines and 5-Aza Treatment

With the exception of A549, HCT116, SKBR3, ZR-75–1, and MCF7, which were purchased from the American Type Culture Collection (http://www.atcc.org), all tumor cell lines were established by us and are deposited at the ATCC or are available upon request [14,15]. Immortalized HBECs were established by us [16,17].

All cancer cell lines were grown in RPMI-1640 medium (Life Technologies [http://www.invitrogen.com]) supplemented with 10% fetal bovine serum. In the present study, unless otherwise indicated, HBECs ectopically express murine *cdk4* and *hTERT*. HBEC lines were grown in KSFM medium supplemented with bovine pituitary extract and recombinant human epidermal growth factor (Gibco [http://www.invitrogen.com]). All cell lines were grown in a humidified atmosphere with 5% CO_2_, at 37 °C. A 50 mM stock solution of 5-aza (Sigma [http://www.sigmaaldrich.com]) was prepared in DMSO and kept at −80 °C until used. Working dilutions were prepared from stock aliquots using DMSO prior to each treatment. Cell lines were incubated in culture medium with 100 nM or 1 μM 5-aza for 6 d, with medium changes on days 1, 3, and 5. For H526, which is nonadherent, cells were agitated with a 200 μl pipette tip in medium containing 5-aza on days 1, 3, and 5. Cells were harvested and total RNA extracted on day 6 using Trizol (Invitrogen).

### Primary Tumors

DNA from resected primary NSCLCs and corresponding normal lung tissue was extracted as previously described [18]. A total of 20 primary lung tumor samples and corresponding nonmalignant lung were randomly selected from a larger panel (*n* = 107) obtained from NSCLC patients who had been treated with curative resectional surgery in The Prince Charles Hospital (Brisbane, Australia) between June 1990 and March 1993. This cohort of patients has been investigated previously for various genetic abnormalities and includes 76 males and 31 females (age range 28–81 y; mean age at diagnosis, 61 y) [18–23]. Of these patients, 61 had stage I disease, 21 had stage II disease, 24 had stage IIIA disease, and one had stage IIIB disease. Histological subtypes included 45 adenocarcinomas, 43 squamous cell carcinomas, 11 adenosquamous carcinomas, four large-cell carcinomas, three atypical carcinoids, and one typical carcinoid. Ninety-eight patients were smokers (mean pack-years, 31), and the rest of patients were never smokers or nonsmokers. Five-year survival data were available on most patients.

Breast tumor DNA was obtained from patients diagnosed with stage IIB or later breast cancer. DNA samples from the University of North Carolina (UNC), the University of Chicago, and Thomas Jefferson University were prepared as previously described [24]. All samples were collected with internal review board approval. Breast tissue sample collection from the University of Texas Southwestern Medical Center at Dallas (Dallas, Texas, United States) was approved by the Institutional Review Board at UT Southwestern Medical Center, and written informed consent was documented for each participant. Random periareolar fine needle aspiration (FNA) was performed as previously described except that the FNA samples were fixed in Preservcyt (Cytyc [http://www.cycyc.com]) [25]. DNA was extracted using the Puregene kit (Gentra Systems [http://www.gentra.com]).

Benign and malignant prostate and colon DNAs were obtained through the UT Southwestern Tissue Resource (UTSTR) overseen by the University of Texas Southwestern Medical Center Institutional Review Board. Tissues were retrieved from the operating room and samples were snap frozen in liquid nitrogen within 30 min off of blood supply. The samples were stored at −80 °C until the DNA was isolated using the Qiagen DNA Isolation Kit (#51306 [http://www.qiagen.com]). The final DNA product was stored in TE buffer at −80 °C until retrieved for sodium bisulfite modification. All DNAs in this group of samples were obtained from patients with stage II or III malignancies.

### Sodium Bisulfite Treatment, Methylation-Specific PCR, and Sodium Bisulfite Sequencing

Sodium bisulfite treatment for the UT Southwestern Medical Center breast FNAs was performed as previously described, using yeast tRNA as a carrier [26]. Sodium bisulfite modification of genomic DNA for the remaining samples and methylation-specific PCR were performed as reported by Herman et al. with some modification to increase sample throughput [27]. We modified the protocol to work in 96-well format as follows: 2 μg of genomic DNA was subjected to sodium bisulfite treatment as before except that samples were incubated in deep-well (1 ml) 96-well plates using a silicon seal (Nunc [http://www.nuncbrand.com]), and reagent concentrations were modified to allow the use of a repeat pipettor (Eppendorf [http://www.eppendorf.com]). An equal volume of membrane-binding solution (Promega [http://www.promega.com]) or 4 M guanidine isothiocyanate (Sigma) was added to the bisulfite reaction after 16 h at 50 °C. The mixture from each well was transferred into the same well on a binding plate held in a 96-well vacuum manifold, and evacuated. Bound DNA was washed three times with 80% isopropanol, then desulfonated in situ with 100 μl of 0.2 N NaOH for 10 min at room temperature. 100 μl of either membrane-binding solution or 4 M guanidine isothiocyanate was added, then evacuated. The desulfonated, bisulfite DNA was washed two more times in 80% isopropanol, and kept under vacuum for 4 min after the last wash to dry the membrane. DNA was eluted into a collection plate with 100 μl of warm (~65 °C), nuclease-free water and further diluted to 250 μl before analysis.

Methylation-specific PCR primers were designed in part by using MethPrimer [28], however substantial modification was necessary in most cases. Of the 132 gene 5-aza induction panel, 45 were selected for methylation analysis because this number enabled accommodation to a 96-well plate format including two control sequences (*TKTL1* and *GAPDH;* total 94 primer sets), and two blank wells for negative controls. Each gene was selected at random from the original 132, and primers were designed using the following criteria: methylation-specific PCR (MSP) primers targeted a region within 250 bp of the annotated transcription start site, where possible (UCSC Genome Browser [http://genome.ucsc.edu] and RefSeq [http://www.ncbi.nlm.nih.gov/RefSeq]), contained three or more CpG sites per primer (most contained four or more), had a 3′-proximal CpG site, and had a predicted annealing temperature of 55 °C or above. If it was not possible to design primers using these criteria, the next gene was selected until a total of 45 was reached. Primers were purchased from Integrated DNA Technologies (http://www.idtdna.com) in 96-well format and diluted to 1 μM. Mixed primers (2 μl each) were added to the corresponding well on prealiquoted 96-well PCR plates (Invitrogen), and 2 μl of diluted bisulfite DNA was added to each well.

PCR conditions and primer sequences may be found in Protocol S1.

PCR products were resolved by electrophoresis using 3% (3:1) agarose in TBE and ethidium bromide. Gels were visualized using a Kodak (http://www.kodak.com) CCD camera and images were collated using Adobe Photoshop CS2 (http://www.adobe.com). Several control gels were run using different combinations of bisulfite DNA, agarose, and running buffers to ensure that the resolving power of the gel was sufficient to identify the appropriately sized bands from primer dimers, which did appear in some cases when no amplicon was present. We were unable to differentiate bands from background for amplicons that were smaller than 90 bp using our final conditions, which precluded use of *GAPDH* as a control. An optically visible band of the appropriate size was called positive for each primer pair.

Sodium bisulfite sequencing was performed using TA cloning (Invitrogen) as described previously [29]. Sequencing data were compiled and analyzed using BiQ Analyzer software, and rendered using a Visual Basic macro in Excel [30].

### Quantitative RT-PCR

Expression of *LOX, NRCAM, BNC1, CCNA1, MAF, ALDH1A3, CTSZ, IRX4, MSX1, KLF11, SERPINB5, TKTL1, GAPDH, r18s,* and *CDKN2A* was analyzed by quantitative real-time RT-PCR. Primers and probes were purchased from Applied Biosystems assay-on-demand, with the exception of *p16,* which was an assay-by-design (Hs00923893_m1) (http://www.appliedbiosystems.com). All samples were run on the Chromo 4 Real Time Detector (MJ Research [http://www.bio-rad.com]) twice, each time in duplicate. We averaged expression of *GAPDH* and *r18s* as internal reference genes to normalize input cDNA. Quantitative real-time reverse-transcriptase-PCR (QPCR) was performed in a reaction volume of 20 μl including 1 μl of cDNA. We used the comparative Ct method to compute relative expression values.

### RNA Quality and Microarray Analysis

RNA from primary lung cancers were obtained as part of collaborations with William Gerald at Memorial Sloan-Kettering Cancer Center (New York dataset) and Chi-Leung Lam and Maria Wong at the University of Hong Kong. All samples were collected with appropriate consent and internal review board approval. Cell line RNA was extracted from cell lines maintained in the Minna laboratory at UT Southwestern Medical Center at Dallas as described above.

The quality of total RNA for all samples was analyzed by formaldehyde gel and/or by capillary electrophoresis on the Experion System (Bio-Rad). Total RNA was labeled and amplified by our genomics core facility, according to manufacturer's instructions (Affymetrix [http://www.affymetrix.com]). cRNA was reanalyzed after labeling to ensure optimal amplification for most of the samples.

cRNA was hybridized to U133 Plus 2.0 (~47,000 transcripts) or U133A (~18,400 transcripts) (Affymetrix), and scanned by our microarray core facility (http://microarray.swmed.edu). Expression analysis of microarray data was performed using several algorithms: Robust Multichip Averaging (RMA) [31,32], Microarray Analysis Suite 5.0 (Affymetrix), MATRIX 1.29 (an array analysis program written by GL [unpublished data]; see below), NIH-DAVID [33], Cluster, and TreeView [34].

After scanning, arrays were checked for quality using GCOS (Gene Chip Operating Software) from Affymetrix and then normalized using either RMA or MATRIX 1.29. For log ratio calculations using MAS5 normalization (MATRIX 1.29), the only requirement was that the numerator be present (Affymetrix *p*-value < 0.065). Data were then logged and renormalized. For RMA normalization, all data were compiled using RMA Express, or RMA through R or BRBArrayTools.

MATRIX (MicroArray TRansformation In eXcel) is a Microsoft Visual Basic program that allows import of multiple CHP files (saved as text file format) from Affymetrix MicroArray Suite 5.0 into an Excel spreadsheet where median normalization, comparison of arrays using log ratios and t-tests, color display, and hierarchical clustering can be performed. Specifically, expression signals are first log_2_-transformed and color coded such that higher signals are displayed as darker (blue) colors. Absent (high detection *p*-value) signals are optionally coded separately on a gray scale. For comparison of samples or classes of samples, log_2_ ratios (i.e., difference of log_2_-transformed signals) are calculated. If samples are compared, the stronger signals must have a present call (detection *p*-value < 0.05). If classes of samples are compared (as log ratios of the means), the median of the detection *p*-values for the class with the highest mean expression value must be less than 0.05. Two-sample t-tests are further calculated to filter out univariate non-significant differential expression. Hierarchical clustering was performed using average linkage with a Pearson correlation metric. All analyses are performed using extensive gene annotation and all probes are BLAST-verified. MATRIX has not been released, as it is still under development. While this program was used extensively in these studies, all analyses were reproduced using publicly available software. Please contact Luc Girard (Luc.Girard@utsouthwestern.edu) for further details.

### Statistical Methods

For CpG island enrichment analysis, intersect tables between the relevant RefSeq gene lists and CpG island annotations were generated using the Table Browser function at the Genome Browser database (http://genome.ucsc.edu/cgi-bin/hgTables?org=Human&db=hg17&hgsid=73574615&hgta_doMainPage=1). Statistical significance for the resultant data was determined using the χ^2^ method where the expected value for 5′ CpG islands for RefSeq annotations was ~37% based on the May 2006 genome build.

Statistical analysis for the primary tumor gene expression data was based on the significance analysis of microarray (SAM) algorithm implemented through BRB ArrayTools, developed by Richard Simon and Amy Peng Lam at NIH. Statistical significance of the methylation data was determined using the χ^2^ method where appropriate.

Correlations between array and QPCR data were determined using the Pearson correlation coefficient. Cluster analysis was performed using Cluster and TreeView either through BRB ArrayTools or directly. Agreement analysis for biological replicate array data was performed as follows: Affymetrix U133 Plus 2.0 .cel files were normalized using RMA implemented through the “Affy” R package (version 1.8.1) from Bioconductor (http://www.bioconductor.org/packages/bioc/1.7/src/contrib/html). To evaluate the consistency of the most differentially expressed genes from biological replicate experiments, we considered a gene to be in agreement if in both experiments, the gene was up- or down-regulated in the same direction compared to control. The agreement analysis consisted of the following steps: (1) calculate log_2_ for each cell line in each experiment: (expression value of the treated cell)/(expression value of the control cell of RMA-normalized data); (2) select the top 1,000 or 2,000 up- or down-regulated genes from each experiment; (3) extract genes that were common to both replicate experiments (union gene set), i.e., genes that were in the top or bottom 1,000 or 2,000 genes in both experiments; (4) calculate the proportion of genes in common for each union dataset, which yields a point estimate for the proportion of agreement: (# of pairs that move in the same direction)/(# of pairs in the union set); (5) for each dataset obtain 5,000 bootstrap samples drawn with replacement from the original dataset; (6) calculate the median and 95% confidence interval (CI; 2.5% and 97.5%) for the agreement proportion [35]. The total number of genes and expressed sequence tags on the array was 54,675.

Enrichment analysis for gene ontology and chromosomal location was performed using NIH-DAVID (http://david.abcc.ncifcrf.gov/home.jsp), using text files containing accession number lists of Affymetrix probe IDs or GenBank (http://www.ncbi.nlm.nih.gov) accession numbers. Statistical enrichment was determined using a Fisher's exact test in which the null hypothesis was that no difference exists between the number of genes falling into a given ontology in the input list and the genome as a whole [33].

### Comparative Genome Hybridization Array

Cell line DNA was isolated using a phenol/chloroform extraction and ethanol precipitation. Each cell line was fingerprinted prior to analysis to ensure that the cell lines were properly identified. Comparative genome hybridization array (aCGH) were performed as previously reported [16,36].

## Results

### Standardizing 5-Aza Treatment for HBECs and Cancer Cell Lines

**Figure 1:**
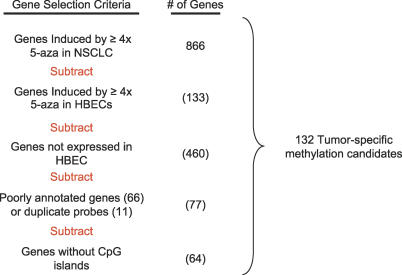
Strategy Used to Identify Methylation Candidates by Gene Expression Microarray NSCLC and HBEC lines were treated with 5-aza and compared to controls (DMSO). We subtracted transcripts induced at least 4-fold in HBEC (*n* = 133) from the total number induced at least 4-fold in two of seven NSCLC lines (866 loci of 47,000 total), since methylation of these genes is unlikely to be tumor specific. For practical purposes we removed genes that were not expressed in HBEC (*n* = 460), were duplicate probes (*n* = 11), or had poor annotations (*n* = 66). Finally we excluded genes without identifiable 5′ CpG islands (*n* = 64). The number of genes subtracted from the total induced ≥4-fold in two of seven NSCLC cell lines (*n* = 866) is indicated next to each description in parentheses. We used the percentage of transcripts associated with 5′ CpG island as a measure of enrichment for the major steps in the filtering process. 37% of all RefSeq transcripts contain 5′ CpG islands; 55% of the 866 5-aza–induced transcripts had 5′ CpG islands; 73% of the final 196 genes had CpG islands. Statistical analysis of these data appears in Table 5.

**Figure 2:**
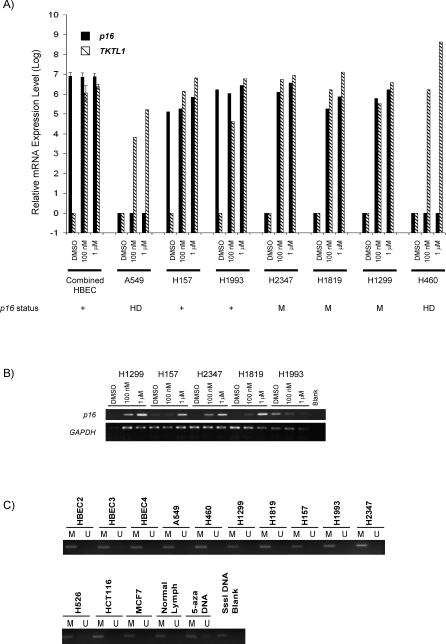
5-Aza Treatment Induces Genes Silenced by Promoter Methylation in HBEC and NSCLC Cell Lines (A) QPCR for *p16* and *TKTL1* in HBEC and NSCLC. Solid bars are *p16* and cross-hatch bars are *TKTL1*. Data are normalized, relative mRNA expression levels according to the 2^ΔΔCt^ method. HBEC2, 3, and 4 had similar profiles and were combined; data are averages and error bars are ± standard deviation. *p16* status is indicated below each cell line; +, expressed; HD, homozygous deletion; M, methylated. (B) RT-PCR for *p16* in the indicated cell lines. *GAPDH* is a loading control. (C) Methylation-specific PCR for *TKTL1* in the indicated samples shows complete methylation in all samples examined, both methylated (M) and unmethylated (U). SssI in vitro-methylated DNA was used as a positive control for the methylated primers, and 5-aza-treated DNA was a positive control for the unmethylated primer sets (for PCR conditions and primer sequences see Protocol S1).

To analyze the gene expression changes associated with loss of promoter methylation in lung cancer cells compared to HBECs, we treated seven NSCLC cell lines (NCI-H460, H1299, H157, H2347, H1819, H1993, and A549) and three HBEC lines (HBEC2, 3, and 4) with low (100 nM) and high (1 μM) doses of 5-aza (Figure 1; Table 1). To determine whether low- and high-dose 5-aza induced genes silenced by promoter methylation in NSCLC cell lines, we performed QPCR for *p16*. We also ran standard reverse transcriptase-PCR for *p16* in several cell lines to ensure that the QPCR primer set did not amplify the alternate splice-form, *p14,* which is expressed in some of these cell lines [37]. We observed induction of *p16* mRNA for both low- and high-dose 5-aza in tumor lines that harbor *p16* promoter methylation (Figure 2A and 2B). Since *p16* could not be used as a positive control for NSCLC lines with homozygously deleted or unmethylated *p16,* we used the universally methylated gene *transketolase-like 1 (TKTL1)* as a positive control for loss of DNA methylation and gene induction. *TKTL1* was induced by 5-aza in all cell lines examined (Figure 2A and 2C).

**Table 1:**
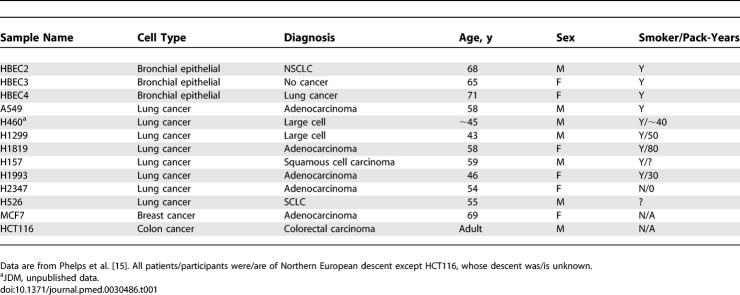
Clinicopathological Features of Cell Lines Used in Microarray Studies

### Microarray Analysis of Gene Expression Changes after 5-Aza Treatment in Lung Cancer Cell Lines

We performed microarray expression profiling on the seven NSCLC and three HBEC cell lines before and after treatment with 100 nM and 1 μM doses of 5-aza, and compared the resultant gene expression profiles. We confirmed our array data in three ways: (1) each cell line was treated with 100 nM and 1 μM doses of 5-aza in a single experiment to confirm array reproducibility and the ability of both doses to induce gene expression (Table 2); (2) biological replication was performed on the three HBEC cell lines 18 months apart on the U133 Plus 2.0 GeneChip, and on four of the seven NSCLC cell lines on the U133A GeneChip, and subsequently on the U133 Plus 2.0 platform (Table 3); (3) QPCR was performed on at least 15 genes in each cell line and at each dose of drug (Table 4; unpublished data).

**Table 2:**
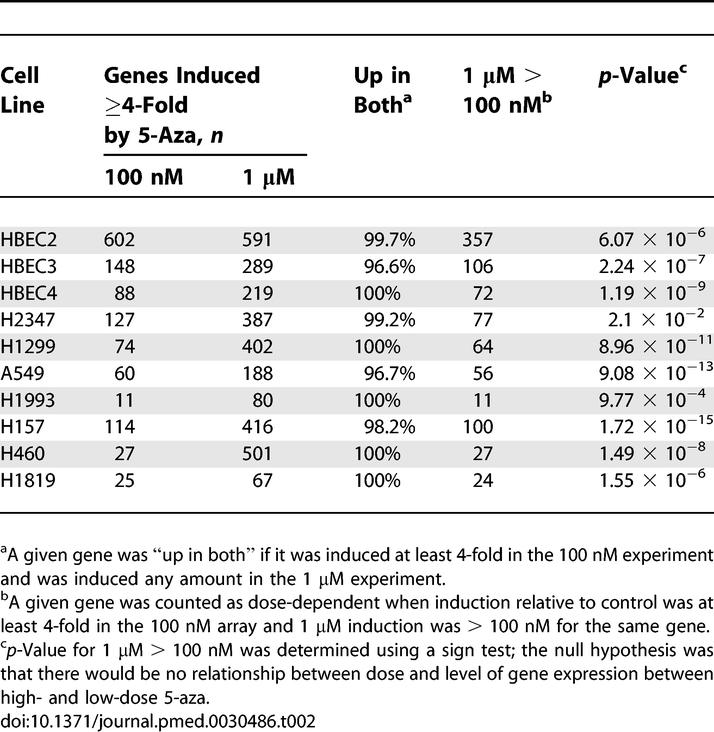
Reproducibility and Dose-Dependence of Gene Induction by 5-Aza-2′-Deoxycytidine

**Table 3:**
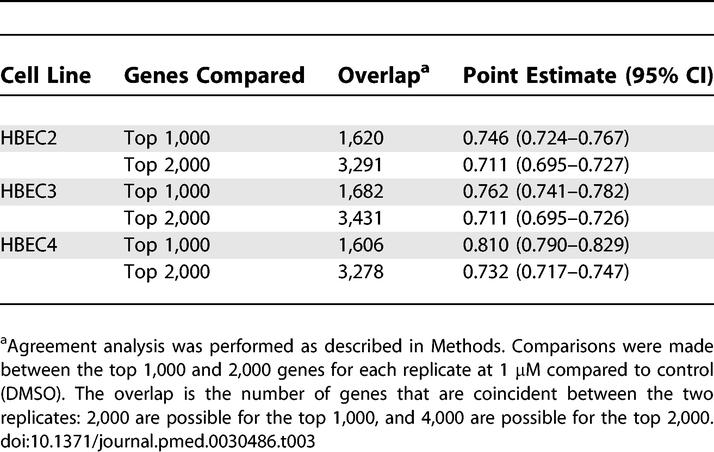
Agreement and 95% CIs for Biological Replicates Performed 18 Months Apart

**Table 4:**
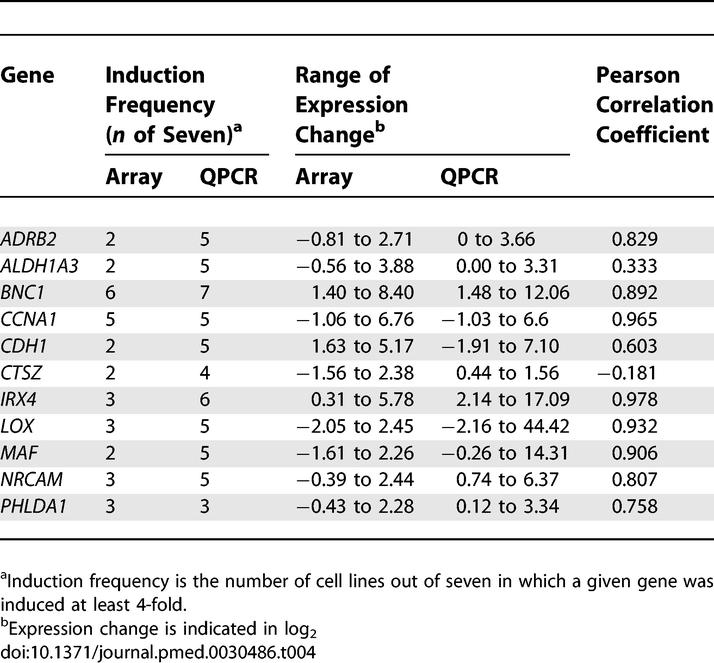
Correlation between Microarray and QPCR Data

**Table 5:**
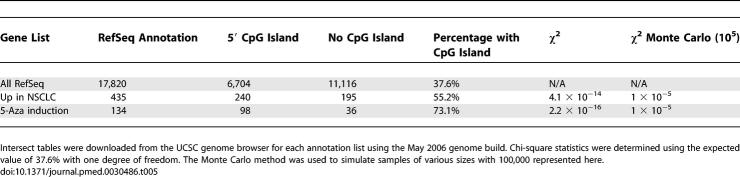
Analysis of CpG Island Enrichment for Genes Induced by 5-Aza in Microarray Experiments

Currently, the thresholds of DNA methylation required to establish gene silencing are unknown, and increasing doses of 5-aza may result in the activation of unrelated gene pathways. However, it has been demonstrated that increasing doses of 5-aza results in increasing levels of demethylation-dependent gene induction [38]. Thus, we used two doses of 5-aza in our array induction experiments. We found a highly significant relationship between both the genes induced in the two treatments and those induced in a dose-dependent manner (Table 2). We determined whether genes were reproducibly inducible by 5-aza over long-term culture by comparing replicates on different types of Affymetrix arrays (U133A GeneChip and U133 Plus 2.0) for four of our seven NSCLC cell lines (A549, H2347, H1299, and H157), as well as data collected on the same type of chip for biological replicates performed 18 months apart on the three HBEC lines. Agreement between HBEC experiments performed 18 months apart was highly significant (Table 3). Gene expression patterns across platforms also correlated well (Pearson correlation coefficients for overlapping gene sets on the two platforms in independent experiments ranged from 0.90 for H157 1 μM to 0.98 for H157 DMSO).

Our analysis of the gene expression profiles of lung cancer cells before and after treatment with 5-aza identified 866 out of 47,000 transcripts that were up-regulated at least 4-fold in two or more lung cancer cell lines (Figure 1). Individually, the cell lines exhibited substantial variations in expression phenotype: H1819 had the fewest (268) genes induced at least 4-fold, whereas H460 had the most (1,100) (Figures S1 and S2). The diversity in gene expression we observed may derive from several factors, including etiology and histopathology (Table 1).

To further validate the induction patterns observed by microarray, we performed QPCR on 15 genes across all cell lines (Table 4). We found that, with the exception of *cathepsin Z (CTSZ),* QPCR analysis correlated well with microarray expression changes. Disagreement between the array and QPCR data for *CTSZ* likely derives from the sensitivity of the Pearson correlation algorithm to small deviations above and below a mean-centered value.

### Isolation of Tumor-Specific Promoter Methylation Candidates

To identify genes that are methylated specifically in cancer cells, we performed similar induction experiments in three HBEC lines. All three HBEC cell lines exhibited changes in gene expression after 100 nM and 1 μM 5-aza treatment (Figure S2A–S2D). In contrast to the cancer cell lines, the HBECs responded similarly to 5-aza treatment. Bioinformatic analysis of the genes induced at least 4-fold in the HBECs suggests that many may be expressed specifically during development or only in certain tissues (Figure S3; Table S1).

Beginning with the 866 transcripts that were induced 4-fold or more in at least two NSCLC cell lines, we excluded 133 that were induced at least 4-fold in HBECs, and we required that a given gene was expressed at a robust median level (MAS5 normalization procedures were used because this method gives an indication of whether a given probe signal is present or absent) in the HBECs with an Affymetrix *p*-value ≤ 0.065. Of the remainder, 460 were excluded on the basis of low (undetectable) expression in the untreated HBEC lines. We further filtered this list of genes by excluding 66 genes without defined 5′ ends or that were otherwise poorly annotated, and 11 that were duplicate probes. This left 196 genes that were induced in the NSCLCs and that met the various filtering criteria.

5-Aza can affect the expression of genes independent of their methylation status [39]. Before restricting the gene set to those with CpG islands, we asked whether our approach had identified a set that was enriched for genes associated with 5′ CpG islands. The null hypothesis was that our selection criteria would make no difference on the frequency of selecting a gene with a CpG island. The expected rate for a RefSeq annotated gene to contain a 5′ CpG island (>500 bp in length) within 2 kb of its transcription start site is ~35% [40]. Based on the March 2006 build, ~37% of the RefSeq 5′-UTR annotations contain 5′ CpG islands within 500 5′ bases. The 866 transcripts we identified on the basis of their induction pattern in NSCLC alone contained 435 RefSeq annotations, while 132 of the 196 transcripts that remained after filtering out genes as described above (Figure 1) had RefSeq annotations. Both of these groups had significant increases in CpG frequency (Table 5).

**Figure 3:**
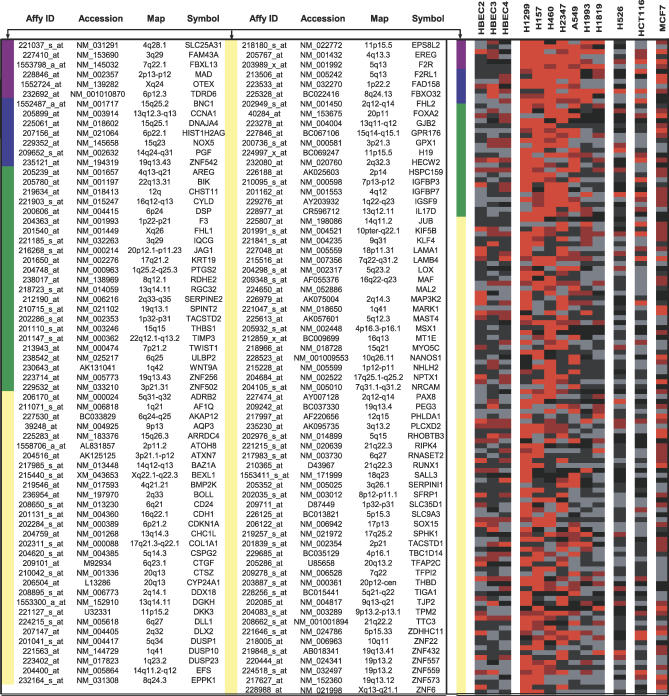
5-Aza-Induced Gene Set in NSCLC, SCLC, Breast, and Colon Cancer Cell Lines Heat map for gene induction across NSCLC and other cancer cell lines as indicated. Data are log_2_ changes between mock-treated and 1 μM 5-aza treatment in each cell line. Bright red indicates 4-fold or greater up-regulation; intermediate red, at least 2-fold induction; grey, less than 2-fold induction; black, no data. The data are ordered from top to bottom according to the frequency of 4-fold induction across the NSCLC cell lines. The vertical colored bars parallel to the heat map represent the frequency of 4-fold induction in the NSCLC 5-aza induction experiments. Affymetrix probe IDs, GenBank accessions, UCSC cytoband alignment, and gene symbols are represented in order from top to bottom with the colored bars from the heat map indicating fold induction; purple indicates five of seven, blue indicates four of seven, green indicates three of seven, and yellow indicates two of seven. The figure layout was borrowed from [66].

On the basis of these data, we examined each of the 196 genes and excluded those that did not have CpG islands defined as larger than 300 bp, a GC content of 55% or more, and an observed versus expected CpG ratio of 0.65 or higher. The remaining 132 transcripts correspond to genes (listed in Figure 3; Table S2) that are candidates for tumor-specific methylation in NSCLC on the basis of their expression pattern in HBECs (i.e., were expressed) and lung cancer cell lines (i.e., were not expressed in several lines), their response to 5-aza in lung cancer cells (induced ≥4 fold), and the presence of a 5′ CpG island (Figure 3).

### Expression Patterns of the 5-Aza Induction Gene Set in Lung Cancer Versus Normal Lung

**Figure 4:**
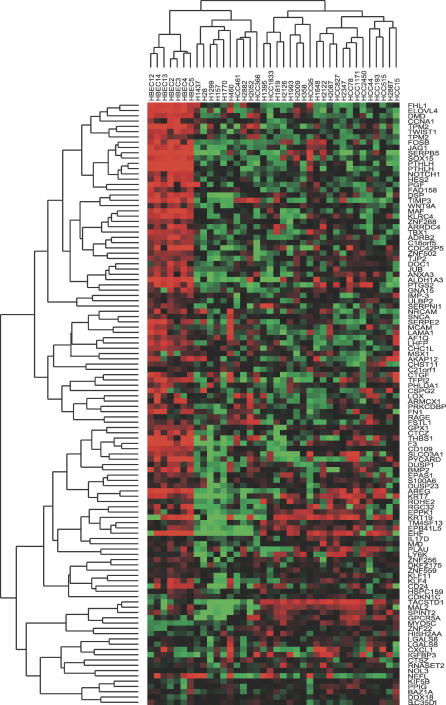
Complete-Linkage Cluster Analysis of 5-Aza-Induced Methylation Candidates in NSCLC and HBECs Analysis was performed on a panel of 31 NSCLC cell lines (U133A and B) and seven HBEC lines (U133 Plus 2.0) with an overlapping gene set (117 genes). Data are mean-centered log_2_ expression values across the samples. Red indicates above the mean; green, below the mean. The 5-aza induction gene set separates cancer from HBEC lines and in most cases these genes are expressed at high levels in HBECs but not in NSCLC.

Although other gene sets were of interest—such as those induced by 5-aza in the HBEC lines, but expressed in the NSCLC panel (i.e., candidate genes that may have undergone tumor-specific promoter hypomethylation and thus function as oncogenes)—in this study we focused on genes that were likely to have undergone tumor-specific promoter hypermethylation leading to inactivation of their expression. We first determined whether our 5-aza induction gene set reflected the gene expression phenotype of a broader set of NSCLC cell lines and HBECs. Using Affymetrix microarray mRNA expression data for NSCLC cell lines (*n* = 31; combined U133A and B chips) and HBEC (*n* = 7; U133 Plus 2.0) lines, we found that all HBEC lines express relatively high levels of these genes, but the lung cancers, while of diverse histologies, express much less (overlap between U133A and B chips with U133 Plus 2.0 included 117 unique genes) (Figure 4). These results suggest that loss of expression of the genes in the 5-aza induction gene set is a common event in NSCLC.

**Figure 5:**
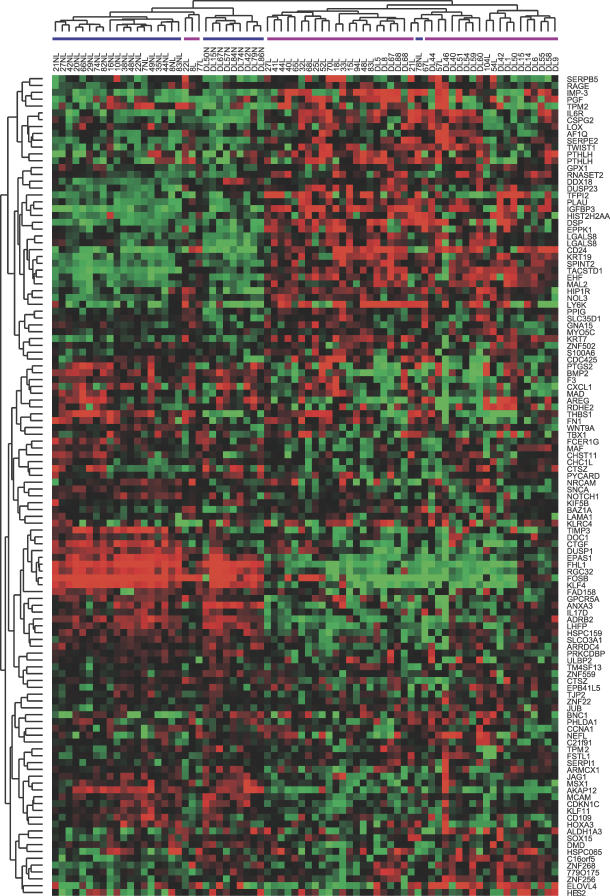
Complete-Linkage Cluster Analysis of 5-Aza-Induced Methylation Candidates in NSCLC and Normal Lung Tissues Panel included 46 primary NSCLC samples and 29 counterpart normal lung tissues. Arrays are median-centered and genes are mean centered and colored as in Figure 4. Blue bar indicates normal lung; purple bar indicates tumor tissue. The 5-aza induction gene set clearly distinguishes cancer from normal. Most genes are expressed at higher levels in normal tissues, although not all.

To determine whether the expression patterns we identified in vitro accurately represent those identified by microarray expression profiling in primary lung cancers, we explored whether the 5-aza induction gene set could distinguish uncultured normal lung from primary lung cancer in two separate microarray datasets. These data are derived from different lung tumor sources (see Methods) collected over a period of several years and comprise expression phenotypes for primary NSCLC (*n* = 45) and counterpart normal lung (*n* = 29), and were randomly selected from a larger panel of array samples. After extracting the relevant probes and filtering the data, we found that the majority of genes were on average expressed at higher levels in the normal samples. While marked gene expression differences between NSCLC and normal lung are to be expected, the 5-aza induction gene set clearly distinguished these phenotypes in our data (Figure 5). Of 117 unique genes in this group, 94 were differentially expressed between tumor and benign tissue based on the SAM algorithm (90th percentile confidence, false discovery rate among the 94 significant genes was 0.11 and the delta value used to identify significant genes was 0.54) (Table S3).

**Figure 6:**
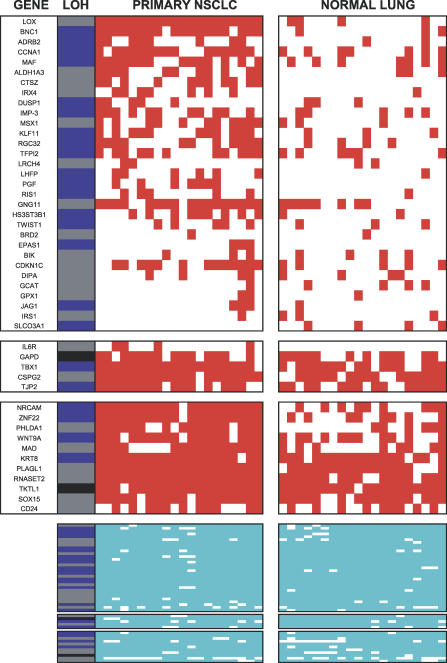
Summary of Methylation-Specific PCR in Matched Primary NSCLC and Adjacent Nonmalignant Tissue Data are color-coded and grouped as follows: red fill indicates positive methylated product; blue indicates positive unmethylated product. Data are grouped as follows: group I, no methylation in either HBECs or PBC DNA; group II, methylation in HBECs but not PBCs; group III, methylation in PBCs. Data are ordered from top to bottom according to the frequency of methylation in primary NSCLC. “GENE” indicates gene symbol; blue bars indicate loss of heterozygosity (LOH; allele loss) (a net [median] copy number change over 31 cell lines was calculated by taking the median signal over all cell lines); grey, no data; black, control primer sequences. Data are presented in the same order in the top (methylated) and bottom (unmethylated) panel.

Tumor-acquired promoter methylation often coincides with allele loss. To determine whether any of the 132 candidate genes were also subject to copy number losses, we analyzed aCGH data for the same panel of NSCLC cell lines that were used for the microarray studies (*n* = 31). Of the 132 genes, approximately half (58/132) had corresponding probes with high-quality data on the Stanford aCGH platform. Of these, 62% (36/58) exhibited a net (median) allele loss across the panel of 31 NSCLC lines (unpublished data; JRP et al., personal communication) (Figure 6). Thus, beginning with 5-aza induction data in lung cancer, we identified 132 genes with 5′ CpG islands that are differentially expressed in primary lung cancer compared to normal lung tissues, many of which are also subject to frequent copy number losses in corresponding NSCLC lines.

### Methylation Analysis of 45 of 132 5-Aza Induction Candidates in Lung Cancer Cells, HBEC Lines, and Normal Peripheral Blood Cells

**Figure 7:**
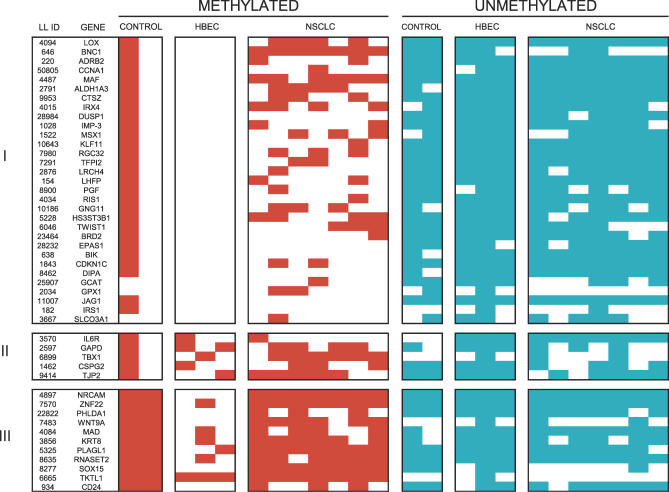
Summary of Methylation-Specific PCR in HBEC and NSCLC Cell Lines From the left, Locus Link ID (LL ID), gene symbol (GENE), in vitro methylated (SssI) DNA mixed with lymphocyte DNA, normal PBC DNA, HBEC lines, NSCLC cell lines, as indicated. Red fill indicates positive methylated product; blue indicates positive unmethylated product. Data are grouped as follows: group I, no methylation in either HBECs or PBC DNA; group II, methylation in HBECs but not PBCs; group III, methylation in PBCs. Data are ordered by the frequency of methylation in primary lung tumors (Figure 6).

To determine whether the genes identified in our screen are methylated in lung cancer cell lines, we designed MSP primer sets (methylated and unmethylated specific) for 45 of 132 candidate genes as well as two control gene primer sets, and tested these on the seven NSCLC lines used for the 5-aza induction studies (for primers, setup, and protocols, see Methods and Protocol S1). As determined by MSP, between 19 and 25 genes out the 45 loci were methylated in any given tumor cell line, whereas at most seven were methylated in the HBECs (Figure 7); several loci were positive for both methylated and unmethylated alleles, consistent with previous studies [41].

As an additional control for tumor-specific methylation, and to determine whether these markers might be useful in a clinical setting, we tested whether any of the genes were methylated in DNA derived from peripheral blood cells (PBCs) of unaffected individuals. This control is important because PBCs are almost always present in biopsy specimens, and the presence of methylation in these cells would preclude use of a given marker for patient screening purposes. Although we found different promoter hypermethylation profiles between different sources of PBCs (unpublished data), in this study a gene promoter was counted as methylated if there was a methylated product in any source of PBCs. By this criterion we found that 11 genes were methylated in at least one PBC source. We grouped the genes according to their methylation patterns as follows: genes with tumor-specific methylation (group I; 31 genes); genes with some methylation in HBECs, but not in normal lymphocyte DNA (group II; five genes); and genes with methylation in PBC DNA (group III; 11 genes).

### Methylation Analysis of 45 of 132 5-Aza Induction Gene Set in Primary Lung Cancers and Normal Lung

It has been suggested that tumor cell lines acquire methylation in culture and as a result may not accurately reflect the methylation patterns of tumors in vivo [5,41]. To address this issue, and to determine whether any of the markers we found were methylated in primary tumor samples, we tested all 45 markers in 20 matched pairs of primary NSCLC and counterpart normal lung tissue (Figure 6). The frequency of methylation in a given tumor ranged from 33 to 17 of the 45 genes. When all genes were included, methylation was significantly more frequent in the matched tumor sample (*p* < 0.001, paired t-test). *Basonucleolin (BNC1)* and *lysyl oxidase (LOX)* were methylated in nearly all of the primary tumors examined, but were not methylated in normal PBCs, and infrequently in normal lung; in comparison, *p16* and *RASSF1A* were methylated in this same NSCLC panel at rates of 30% and 40%, respectively [18]. The appearance of low-level methylation in some normal counterpart tissue may result from field effects and/or tumor cell contamination. Some markers were methylated at high frequency in tumors (>30%; compared to *p16* and *RASSF1A,* 30% and 40%, respectively) and never in matched normal tissue such as *CTSZ* and placental growth factor *(PGF)*.

In general, the methylation frequency of group I genes was similar to that of the cell lines used in this study; where there was frequent methylation in the cell lines, there was frequent methylation in the primary tumors (Figures 6 and 7). Group II and III genes also followed the patterns identified in the cell lines; where methylation was found in the HBECs, methylation was frequent in both primary tumors and matched normal lung. When methylation was detected in normal PBC DNA and/or HBEC DNA, methylation was evident in both primary tumor and normal lung DNA samples (which has PBC contamination). While all of these genes could be involved in lung cancer pathogenesis through promoter methylation and concomitant loss of expression, we focused on the 31 group I genes as being the best candidates for diagnostic markers to avoid genes found to be methylated in normal lung or PBCs.

### Comparison of 5-Aza Induction Gene Expression Profiles in Breast Cancer, Colon Cancer, and Small Cell Lung Cancer Cells

While there was some overlap between genes induced by 5-aza among the NSCLC lines, the predominant pattern we found reflects significant expression differences within the same tissue type (Figure S3). The diversity we observed in NSCLC led us to explore whether other epithelial cancers differ dramatically in their response to 5-aza. We performed the 5-aza induction experiments in breast cancer, colon cancer, and small-cell lung cancer (SCLC) cells using our standard protocol with a minor modification for the nonadherent SCLC cell line (see Methods). When we compared these cell types after 5-aza induction by (SAM) and cluster analysis, we found that although each cell line clustered with itself independent of treatment, SCLC and breast cancer cells, but not the colon cancer cell line HCT116, clustered apart from NSCLC (Figure S4). However, after supervised hierarchical cluster analysis using our final 5-aza induction gene set, tissue-of-origin distinctions were no longer apparent (Figure S5). These data suggest that part of the 5-aza induction response in these cell lines may be independent of tissue-specific gene expression or promoter methylation profiles.

To further explore the finding that 5-aza induction patterns in cancer cell lines may be independent of tissue of origin differences, we compared our dataset to those of Sato et al. [41], who used the Affymetrix U133A chip to examine gene induction patterns after 5-aza treatment in four pancreatic cancer cell lines. The authors reported that 475 genes were up-regulated over 5-fold in at least one cell line. Of these 475 genes, 203 were also up-regulated in at least one of our cell lines, with 127 up-regulated in two or more (Table S4). Bioinformatic analysis of the overlapping gene set between the Sato et al. and our data indicates some highly significant similarities in the position of the genes induced by 5-aza in lung and pancreas (Table S5), but unfortunately robust statistical analysis of this finding was not possible due to the unavailability of the raw data and differences in experimental setups. Multiple genes in two chromosomal regions, Xp11.2–11.4 and 6p21.3, were induced in both types of cell lines, and, based on the gene density in these genomic regions, each enrichment was highly significant (*p* = 3.01 × 10^−9^ and *p* = 1.01 × 10^−7^, respectively, Fisher's exact test).

Next we analyzed the expression pattern of the 5-aza induction gene set across a panel of breast cancer cell lines and found that for the 5-aza induction panel (by average linkage cluster analysis), most of the lung cancer cells and approximately half of the breast cancers fall into a major cluster distinct from the remaining breast cancer cells and the immortalized HBECs, which form their own tight cluster with a minimum Pearson correlation coefficient of greater than 0.7 (Figure S6). These data suggest that tumor-specific, rather tissue-specific, gene expression patterns are the predominant factor driving the clustering algorithm for the 5-aza induction gene set. To confirm these findings, we examined 15 of the genes found to be frequently induced by 5-aza and methylated in NSCLC in six breast cancer cell lines (HCC3153, HCC1143, HCC1937, SKBR3, ZR-75–1, and MCF7) and found nearly all to be induced by 5-aza in these cells (Table 4; Figure S7). The overlap we found in the gene induction patterns between NSCLC, SCLC, breast, and colon cancer cells in our 5-aza induction microarray experiments, those in our breast cancer cell line panel, and those previously reported in pancreatic cancer cells suggested to us that some of these genes may be methylated in breast cancer and other cancers [42].

### Methylation Analysis of Select Genes in Primary Breast Cancer and Counterpart Normal Tissue

**Figure 8:**
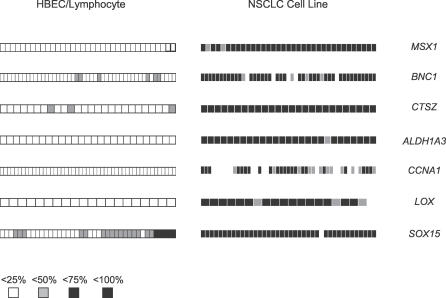
Summary of Sodium Bisulfite Sequencing for Seven Genes in PBCs, HBECs, and NSCLC Cells Between eight and 20 clones were sequenced for each locus in each cell type. Sequencing primers were designed to flank the MSP priming sites and do not include any CpG sites, with the exception of *BNC1,* which we were not able to amplify outside of the MSP priming sites for cells that harbored methylation. There was no amplification of the methylated primer set in HBECs or PBCs, and no amplification of the unmethylated primer set in the NSCLC cell lines examined. One some occasions the methylated primer set for *BNC1* amplified a 289 bp amplicon from an unrelated locus on Chromosome 1. The sequence corresponds to a CpG island in an intronless gene *(GPR25)* that was heavily methylated in tumors. The unmethylated primer set did not amplify this sequence. Each box represents a composite of clones for that CpG site. Open boxes indicate 0%–25% methylation; light grey, 26%–50%; dark grey, 51%–75%; black, 76%–100% methylation. Raw data are available in Figures S8–S14. Primers and PCR conditions are available upon request.

We selected eight of 15 markers that were induced by 5-aza in both lung and breast cancer cells for analysis in primary breast tumor material. Of the primary breast tumors used in this study, 23 form part of a large dataset used in several studies in which fundamental histological and phenotypic differences were defined between subtypes of ductal breast carcinomas [43]. The DNA from these samples was derived from bulk tumor specimens upon surgical resection from the primary tumor site, metastatic sites, or at autopsy. With one exception, all of these tumor specimens were stage IIB or later. We found that among the eight genes tested in 23 breast carcinomas, seven were frequently methylated (60%–90%) (Figure 8). These breast cancer samples did not have counterpart normal tissue.

**Figure 9:**
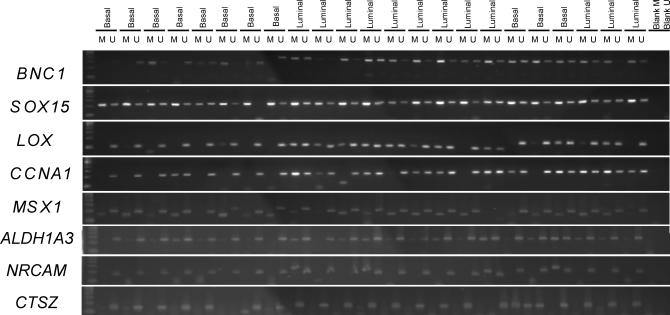
MSP for Indicated Genes in Ductal Breast Carcinoma DNA for Samples Obtained from UNC The basal phenotype is based on gene expression profiles demonstrated previously and is characterized by the absence of estrogen receptor and a poor prognosis. Other samples are characterized as luminal. Visible bands corresponding to the appropriate size were counted as positive. 100 bp ladder is at far left. M, methylated product; U, unmethylated product.

To address whether methylation for these eight genes was detectable in benign breast tissue, an additional 14 tumor samples that have matched benign material were examined (see Methods); these samples are primarily early-stage tumors (stage IIB or earlier) collected upon surgical resection of the primary tumor. The counterpart benign tissue was collected by FNA in the ipsilateral breast (except where indicated) and have not been described previously. As with the later stage breast tumor samples methylation was common, although overall there was more methylation in the more advanced tumor stage group. Only *SOX15* exhibited frequent methylation in benign breast material (Figures 8 and 9; Table 6).

**Table 6:**
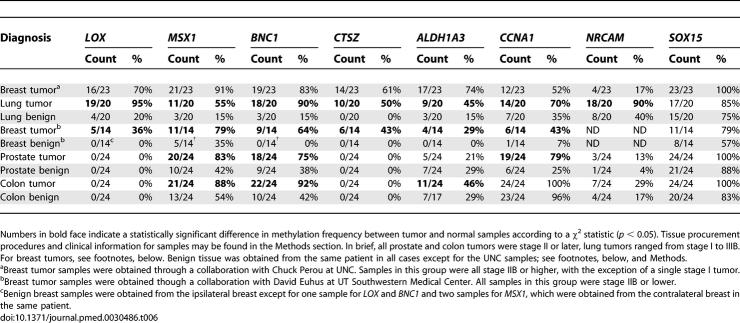
Frequency of Promoter Hypermethylation for Eight Genes as Determined by MSP for Indicated Tumors

Methylation-specific PCR, while robust, is extremely sensitive and can detect methylated sequences in the presence of large amounts of unmethylated DNA. We used sodium bisulfite DNA sequencing to confirm that the MSP primer sets used in these studies amplified the appropriate target sequences and that these sites were bona fide hypermethylated CpG islands. We designed primers that flank the MSP priming sites for the eight genes examined and then cloned and sequenced PCR products from bisulfite-treated HBEC and/or lymphocyte DNA and tumor cell DNA. Between eight and 20 subclones from each selection plate for each cell type and gene were analyzed. With the exception of *NRCAM,* all sequences were heavily methylated in the tumor cells but not in HBEC or PBC DNA (Figures S8–S14 and 8). Based on these data, and its infrequent methylation in breast cancer, we excluded *NRCAM* from subsequent analyses.

### Examination of the Methylated Gene Set in Matched Pairs of Colon and Prostate Cancers and Companion Benign Epithelium

**Figure 10:**
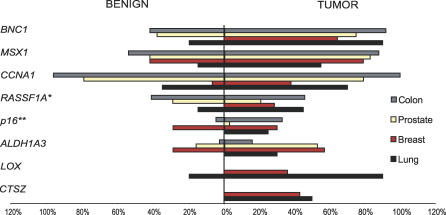
Histogram for Methylation Frequency of Indicated Genes in Prostate, Breast, Lung, and Colon Cancer and Companion Normal Tissue MSP data for indicated genes in breast (*n* = 14; red bars), lung (*n* = 20; black bars), prostate (*n* = 24; pale yellow bars), and colon (*n* = 24; grey bars) tumors and benign tissue (see Methods). Only samples with matching benign and tumor tissue are represented in the histogram. Gels were run and scored as above. *SOX15* was omitted from this figure for clarity. Data for *RASSF1A* were obtained from [17,24,45,46]; data for *p16* were obtained from [17,43,44,46].

Tumor-specific promoter hypermethylation is often also tissue-specific. To explore whether the seven genes *(BNC1, LOX, ALDH1A3, MSX1, CCNA1, CTSZ,* and *SOX15)* we identified in the previous section were methylated in other tissues besides breast and lung, we examined an independent set of primary colon and prostate cancers and their matched normal tissues. For comparative purposes we included methylation data for *p16* and *RASSF1A* for all tumor types examined (Figure 10; Table 6). Data for *RASSF1A* and *p16* are derived from published work as annotated in the legend for Table 6 [18,25,42,44–47].

*BNC1, MSX1,* and *CCNA1* were frequently methylated in all four tumor types. However, *CCNA1* exhibited significant methylation in benign prostate and colon tissues. This suggests that *CCNA1* may undergo tissue-specific methylation during cellular differentiation in certain tissues but not others. *BNC1* and *MSX1* showed high sensitivity and specificity for tumors when compared to benign counterpart tissues (estimated values [95% CIs]: 0.81 [0.75 to 0.86] and 0.67 [0.60 to 0.75], respectively). For *BNC1* and *MSX1,* both prostate and colon benign tissues did have some methylation, but the pattern was different from *CCNA1*. *ALDH1A3* was specifically methylated in all tumor types, albeit less frequently than *BNC1* or *MSX1,* showing the highest sensitivity in breast and prostate and highest specificity in lung. *LOX* and *CTSZ* methylation was restricted to lung and breast tumors, and in both cases were highly specific. *SOX15* was methylated in most benign tissues and has been omitted from the histogram for clarity.

## Discussion

We used global gene expression profiling (47,000 transcripts) of seven lung cancer cell lines before and after treatment with 5-aza to identify genes that were significantly up-regulated by this treatment. We performed similar experiments in three newly available immortalized HBEC lines to identify genes whose expression was selectively lost in lung cancer, expressed in normal lung epithelium, but inducible by 5-aza treatment. To our knowledge the use of these cells as part of a global methylation induction screen has not been described previously. We applied a series of biological filters to extract a list of methylation candidates, and statistical analyses of the major steps in this process suggested that successive lists were enriched for genes with 5′ CpG islands. Only those genes that were induced in more than one lung cancer and had well-defined CpG islands in their putative promoter regions were selected. This filtering process led us to identify 132 candidate genes, 45 of which we investigated in detail in the current study.

The large majority of the 132 genes we have identified have not been described to undergo tumor-specific promoter hypermethylation and expression of these genes distinguishes primary lung cancers from normal lung in the same patient. While many genes are probably methylated—perhaps at random—during carcinogenesis, we found that 31 of the 45 genes studied here undergo tumor-specific methylation in multiple primary lung cancers. We studied eight of these 45 genes in a panel of 105 primary tumors from NSCLC, breast, colon, and prostate cancers and 82 histologically normal companion tissues, which showed that these genes undergo methylation in common epithelial cancers. Frequent methylation of specific genes in multiple independent cancers strongly suggests but does not prove that these genes are functionally relevant to cancer pathogenesis.

One goal of this study was to identify new genes involved in tumor-specific methylation for follow-up functional analysis. To this end, our screen uncovered some well-established methylation markers that have tumor suppressor activity, including *TIMP3, CDH1,* and *SFRP1,* but missed others such as *p16* and *RASSF1A*. That we missed some of the classical methylation markers highlights a limitation of current microarray technology: commercial arrays cannot always discriminate between alternative splice forms of genes; both *p16* and *RASSF1* have constitutively expressed alternative isoforms that can hybridize to probes specific for these loci. Since both genes have expressed isoforms *(p14* and *RASSF1C)* that differ only in their 5′ regions, none of the probes specific to these genes detected differences in expression. This limitation means that we have probably missed isoforms of genes that are subject to tumor-specific methylation, but that are part of an active transcription locus.

Most of the genes identified in this study are novel methylation candidates in NSCLC, although methylation of some of them has been described in other tissues. *LOX* was frequently methylated in our panel of cell lines and NSCLC tumors, and was recently shown to be methylated in gastric cancers [48]. *CCNA1* was shown to be methylated in head and neck cancers and was inversely correlated with *p53* mutation [49]. In our study, *CCNA1* was methylated in A549, which has wild-type *p53,* but was not methylated in NSCLC cells with mutant *p53*. Loss of *dual-specificity phosphatase I (DUSP1)* expression as determined by immunohistochemistry inversely correlates with increasing malignancy of prostate cancers, and methylation of its promoter appears to be an early event in this disease [50]. In another recent report, *tissue factor pathway inhibitor 2 (TFPI2)* methylation was used as part of a six-gene panel to screen for cancer in pancreatic juice specimens [51]. Promoter methylation of the transcription factor *TWIST1* has been described in several reports and is frequent in neuroblastoma, cervical, and breast cancers, although high expression of *TWIST1* seems to be necessary for breast cancer metastasis [52–55]. The proapoptotic *BCL2* family member *BIK* was identified in a global screen for promoter methylation in multiple myeloma using restriction landmark genomic scanning [56].

Our data suggest that some genes, such as *CCNA1,* undergo both tissue-specific and tumor-specific methylation. Tissue-specific promoter hypermethylation arises in response to both extrinsic and intrinsic signals during cellular differentiation and may account for the distinctive methylation pattern we observed for this particular cyclin [57]. The biological basis of frequent tumor-specific hypermethylation in multiple tissues coincident with tissue-specific methylation in another tissue is unknown. However, two well-characterized tumor suppressors, *p16* and *RASSF1A,* exhibit similar tumor-specific and tissue-specific promoter methylation profiles; *p16* methylation is frequently observed in benign breast tissue, even in young women, and *RASSF1A* promoter hypermethylation is observed in benign liver and colonic epithelium [45,58]. Thus, the presence of promoter methylation in selected normal tissues does not exclude a gene from being an important tumor suppressor. Nevertheless, the information on such methylation is important for clinical applications.

Another pattern of promoter hypermethylation evident in our data, exemplified by *LOX* and *CTSZ,* is characterized by frequent but exclusive methylation in certain tumor types. According to data available through various online databases such as Genecard (Weizmann Institute [http://genome-www.stanford.edu/genecards/index.shtml]) and Source (Standford University [http://genome-www5.stanford.edu/cgi-bin/source/sourceSearch]), both *LOX* and *CTSZ* are widely expressed. Both genes also have several homologs that may be partially redundant, or they may have tissue-specific functions important to tumorigenesis in breast and lung, but not in prostate or colonic epithelium. Several other genes exhibit a similar, restricted methylation profile, such as *breast cancer 1, early onset (BRCA1)* in breast and ovarian tumors, and *glutathione S-transferase pI (GSTP1)* in liver and prostate cancers [59,60]. Genes that are methylated with high frequency and specificity only in certain tumors would be valuable in the development of a promoter hypermethylation profile to screen for several cancers in parallel.

Perhaps the most important profile identified in this study is that of tumor-acquired methylation involving the four most common epithelial tumors. When all matched tumors were combined, *BNC1* and *MSX1* were both highly sensitive and specific for tumor detection. As yet, relatively few loci have been identified that exhibit frequent (>50%), tumor-specific methylation across several types of malignancies. Several genes exhibit frequent methylation in NSCLC and other tumor types, such as the tumor suppressor gene *adenomatosis polyposis coli (APC)* or *retinoic acid receptor beta (RARβ),* but these genes are often also methylated in counterpart benign tissue, especially in tumors for which field effects are common, such as NSCLC [18,61]. The identification of more loci like *BNC1* and *MSX1* will be an essential element to developing a promoter hypermethylation profile for the early detection of human cancer.

Relatively few tumor-specific lesions occur with significant frequency in all types of tumors, with the important exceptions of *p53* mutation, genomic instability, and constitutive reactivation of telomerase [62–64]. The wealth of data available in the scientific literature suggests that aberrant DNA methylation may be another key contributor to cellular transformation. The frequency and diverse patterning of tumor-specific promoter methylation in our panel of lung, colon, prostate, and breast carcinomas, coupled with the findings recently reported by others, indicate that tumor-acquired promoter hypermethylation patterns are nonrandom [6,65]. While it is possible that random methylation events are ongoing in cancer cells, that some genes are so frequently methylated across different tumors but not in adjacent normal tissues suggests to us that something about their function or primary sequence makes them particularly susceptible to aberrant promoter hypermethylation during cellular transformation.

By contrasting the genome-wide changes in gene expression of normal and lung cancer cells, we were able to gain insight into the complexity of the methylation program required for cells to become fully malignant. Even though we began with a highly structured, organ-specific screen, by applying successive biological and statistical filters we identified several genes with exceptionally high methylation frequencies and tumor specificity in primary lung and breast tumors. Several of these genes also show significant methylation in colon and prostate tumors, but not in counterpart benign tissues. We conclude that, while tumors differ in their molecular phenotypes and pathogenesis, the pathways they follow toward malignancy may be similar and may be reflected in the methylation programs they engage. If true, it follows that identifying the common pathways tumor cells use and the methylation profiles they impart may be useful to exploit for early diagnosis or therapeutic intervention.

## Supporting Information

Figure S1 Scatter Plots Showing Gene Expression Changes after 5-Aza Treatment in NSCLC (A) H157: 2-fold changes show more than 4,000 genes up-regulated, with similar numbers down-regulated.
(B) H1819: 2-fold changes show fewer than 1,000 genes regulated both up and down in this cell line.
(C) H460: 4-fold changes.
(D) H1819: 4-fold changes. Red dots indicate up-regulated genes; green indicates down-regulated genes.
(136 KB PDF)

Figure S2 Scatter Plots Showing Gene Expression Changes in HBEC Cell Lines after 5-Aza Treatment (A) HBEC2: 2-fold changes.
(B) HBEC3: 2-fold changes.
(C) HBEC4: 2-fold changes.
(D) Average 2-fold changes for all three HBECs
(153 KB PDF)

Figure S3 Complete-Linkage Cluster Analysis of 5-Aza-Induced Genes (before Filtering) in HBEC and NSCLC Cell Lines (U133 Plus 2.0) Used for This Study (9 KB PDF)

Figure S4 Complete-Linkage Cluster Analysis of 5-Aza-Induced Genes (before Filtering) in HBEC, NSCLC, Breast, SCLC, and Colon Cancer Cell Lines (U133 Plus 2.0) Used for This Study (10 KB PDF)

Figure S5 Complete-Linkage Cluster Analysis of 5-Aza-Induced Genes (132-Gene 5-Aza Induction Set) in HBEC and NSCLC Cell lines (U133 Plus 2.0) Used for This Study (10 KB PDF)

Figure S6 Comparison of the Gene Expression Profiles for the 5-Aza Induction Gene Set in HBEC, NSCLC, and Breast Cancer Cell Lines (A) Heat map showing relative gene expression for the 132 5-aza-induced gene set as well as those that passed all criteria except that they lacked a CpG island.
(B) Cluster analysis of breast, NSCLC, and HBEC lines using the gene set from (A).
(50 KB PDF)

Figure S7 Histogram of QPCR Data for 5-Aza-Induced Gene Expression Changes in Breast Cancer Cell Lines (17 KB PDF)

Figure S8 Sodium Bisulfite Sequencing Results for *BNC1* Promoter Region in NSCLC Cell Lines Compared to HBECs and Normal PBCs (Mixture) (14 KB PDF)

Figure S9 Sodium Bisulfite Sequencing Results for *MSX1* Promoter Region in NSCLC Cell Lines Compared to HBECs and Normal PBCs (Mixture) Found at doi:10.1371/journal.pmed.0030486.sg009 (25 KB PDF)

Figure S10 Sodium Bisulfite Sequencing Results for *ALDH1A3* Promoter Region in NSCLC Cell Lines Compared to HBECs and Normal PBCs (Mixture) Found at doi:10.1371/journal.pmed.0030486.sg010 (20 KB PDF)

Figure S11 Sodium Bisulfite Sequencing Results for *LOX* Promoter Region in NSCLC Cell Lines Compared to HBECs and Normal PBCs (Mixture) (19 KB PDF)

Figure S12 Sodium Bisulfite Sequencing Results for *CTSZ* Promoter Region in NSCLC Cell Lines Compared to HBECs and Normal PBCs (Mixture) (19 KB PDF)

Figure S13 Sodium Bisulfite Sequencing Results for *SOX15* Promoter Region in NSCLC Cell Lines Compared to HBECs and Normal PBCs (Mixture) (26 KB PDF)

Figure S14 Sodium Bisulfite Sequencing Results for *CCNA1* Promoter Region in NSCLC Cell Lines Compared to HBECs and Normal PBCs (Mixture) (30 KB PDF)

Protocol S1 Primer Sequences and PCR Conditions for MSP (54 KB XLS)

Table S1 Bioinformatic Analysis of 5-Aza-Induced Genes in HBECs Gene expression in immortalized cells is significantly affected by 5-aza treatment.
(17 KB XLS)

Table S2 Raw Data for Figure 3
Raw data underlying the heat map in Figure 3.
(114 KB XLS)

Table S3 SAM Analysis of 5-Aza Gene Set in Primary Tumors SAM analysis of 5-aza-induction gene set in primary tumors and companion normal lung.
(159 KB XLS)

Table S4 Overlapping Gene Set Between the Current Study and a Previous Study Comparison between the present study and a previously published set of microarray experiments using pancreatic cancer cell lines [41].
(204 KB XLS)

Table S5 Analysis of Overlap between the Current Study and a Previous Study Bioinformatic analysis of overlapping gene set between Sato et al. [41] and the current study.
(26 KB XLS)

### Accession Numbers

The microarray data for the 5-aza induction experiments are deposited at the GEO database (http://www.ncbi.nlm.nih.gov/projects/geo/) under the accession ID GSE5816.

## Abbreviations

5-aza: 5-aza-2′-deoxycytidine
aCGH: comparative genome hybridization array
CI: confidence interval
FNA: fine-needle aspiration
HBEC: human bronchial epithelial cell
MSP: methylation-specific PCR
NSCLC: non-small cell lung cancer
PBC: peripheral blood cell
QPCR: quantitative reverse transcriptase-PCR
RMA: robust multichip averaging
SAM: significance analysis of microarray
SCLC: small cell lung cancer
