# Supplementary material for: A Genome-Wide Screen for Promoter Methylation in Lung Cancer Identifies Novel Methylation Markers for Multiple Malignancies
Source: PLoS Med. 2006 Dec 26;3(12):e486. doi: 10.1371/journal.pmed.0030486 (PMC1716188; doi:10.1371/journal.pmed.0030486)
Supplement: Supplementary file 1 [file pmed.0030486.sg001.pdf]

Fig. S1

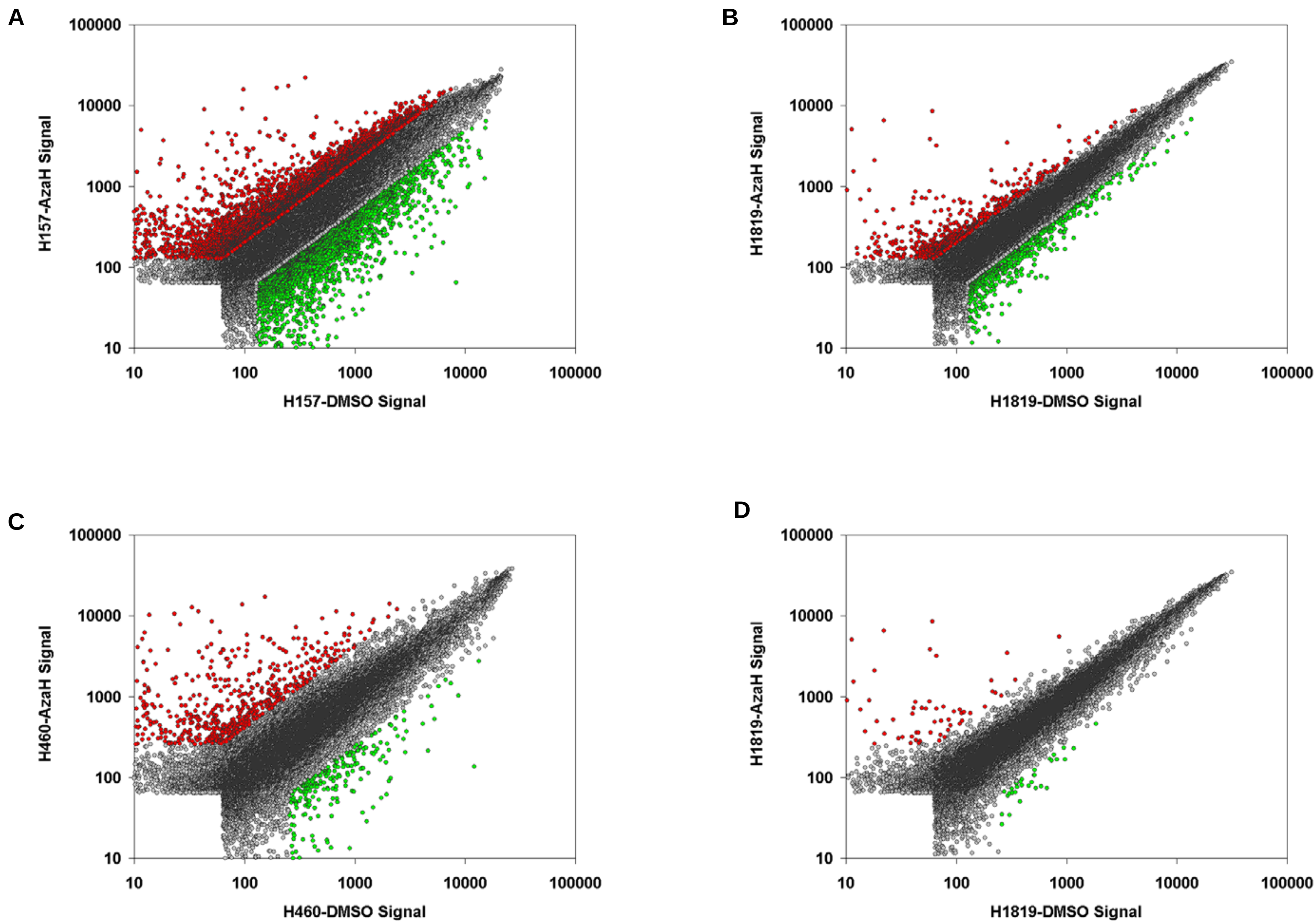
A
B
C
D
100000
10000
1000
100
10
H157-AzaH Signal
H157-DMSO Signal
H1819-AzaH Signal
H1819-DMSO Signal
H460-AzaH Signal
H460-DMSO Signal
