# Supplementary material for: A Genome-Wide Screen for Promoter Methylation in Lung Cancer Identifies Novel Methylation Markers for Multiple Malignancies
Source: PLoS Med. 2006 Dec 26;3(12):e486. doi: 10.1371/journal.pmed.0030486 (PMC1716188; doi:10.1371/journal.pmed.0030486)
Supplement: Supplementary file 2 [file pmed.0030486.sg002.pdf]

Fig. S2

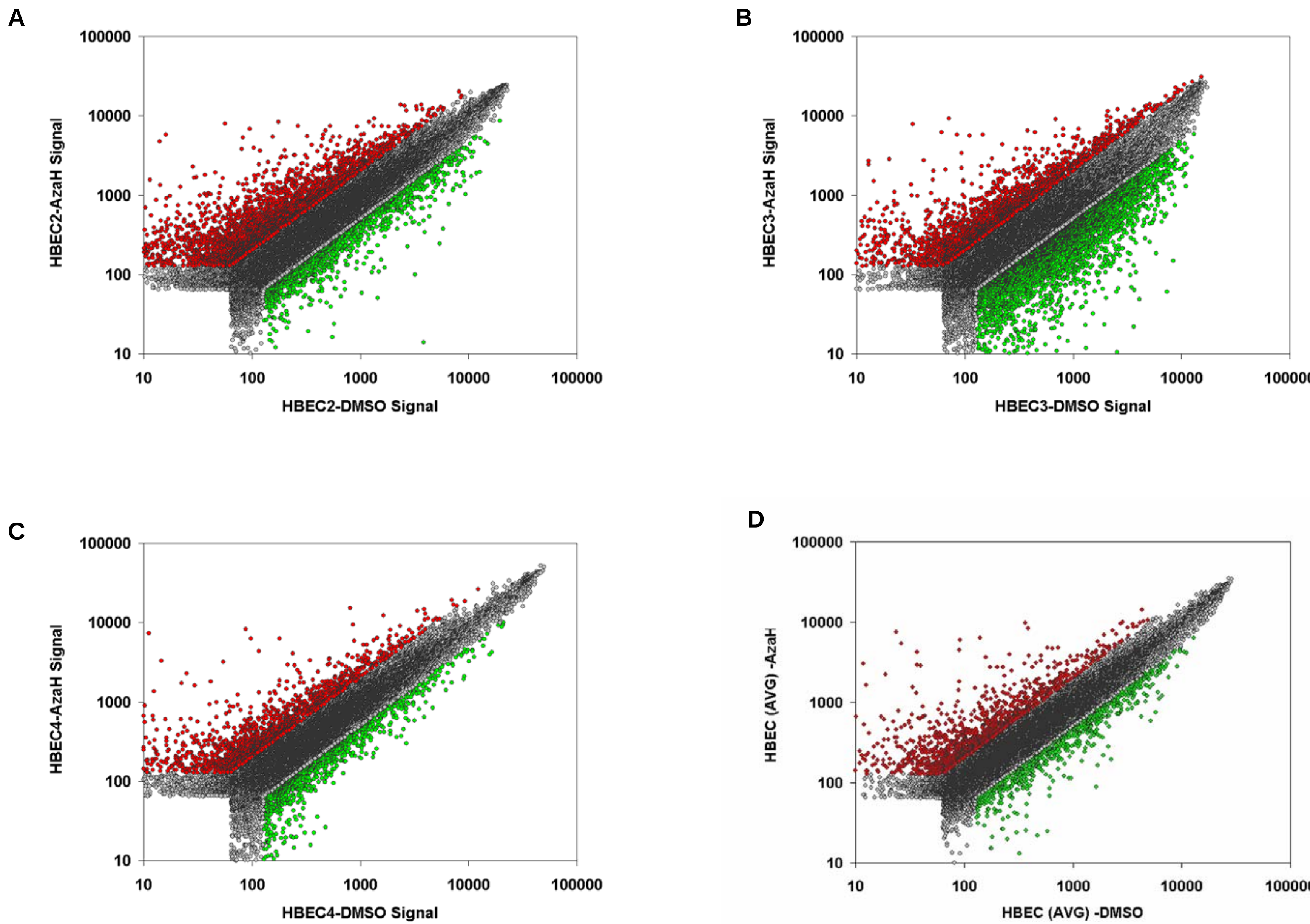
A
B
C
D
100000
10000
1000
100
10
HBEC2-AzaH Signal
HBEC2-DMSO Signal
HBEC3-AzaH Signal
HBEC3-DMSO Signal
HBEC4-AzaH Signal
HBEC4-DMSO Signal
HBEC (AVG) -AzaH
HBEC (AVG) -DMSO
