# Supplementary material for: A Genome-Wide Screen for Promoter Methylation in Lung Cancer Identifies Novel Methylation Markers for Multiple Malignancies
Source: PLoS Med. 2006 Dec 26;3(12):e486. doi: 10.1371/journal.pmed.0030486 (PMC1716188; doi:10.1371/journal.pmed.0030486)
Supplement: Supplementary file 4 [file pmed.0030486.sg004.pdf]

Fig. S4

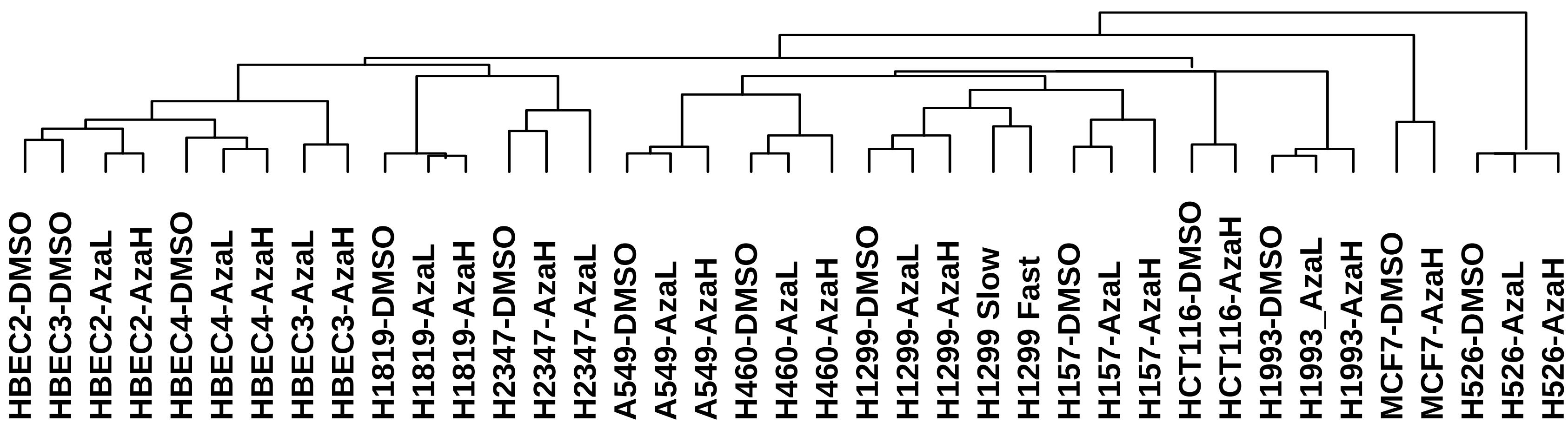
HBEC2-DMSO
HBEC3-DMSO
HBEC2-AzaL
HBEC2-AzaH
HBEC4-DMSO
HBEC4-AzaL
HBEC4-AzaH
HBEC3-AzaL
HBEC3-AzaH
H1819-DMSO
H1819-AzaL
H1819-AzaH
H2347-DMSO
H2347-AzaH
H2347-AzaL
A549-DMSO
A549-AzaL
A549-AzaH
H460-DMSO
H460-AzaL
H460-AzaH
H1299-DMSO
H1299-AzaL
H1299-AzaH
H1299 Slow
H1299 Fast
H157-DMSO
H157-AzaL
H157-AzaH
HCT116-DMSO
HCT116-AzaH
H1993-DMSO
H1993_AzaL
H1993-AzaH
MCF7-DMSO
MCF7-AzaH
H526-DMSO
H526-AzaL
H526-AzaH
