# Supplementary material for: A Genome-Wide Screen for Promoter Methylation in Lung Cancer Identifies Novel Methylation Markers for Multiple Malignancies
Source: PLoS Med. 2006 Dec 26;3(12):e486. doi: 10.1371/journal.pmed.0030486 (PMC1716188; doi:10.1371/journal.pmed.0030486)
Supplement: Supplementary file 6 [file pmed.0030486.sg006.pdf]

Fig. S6 A

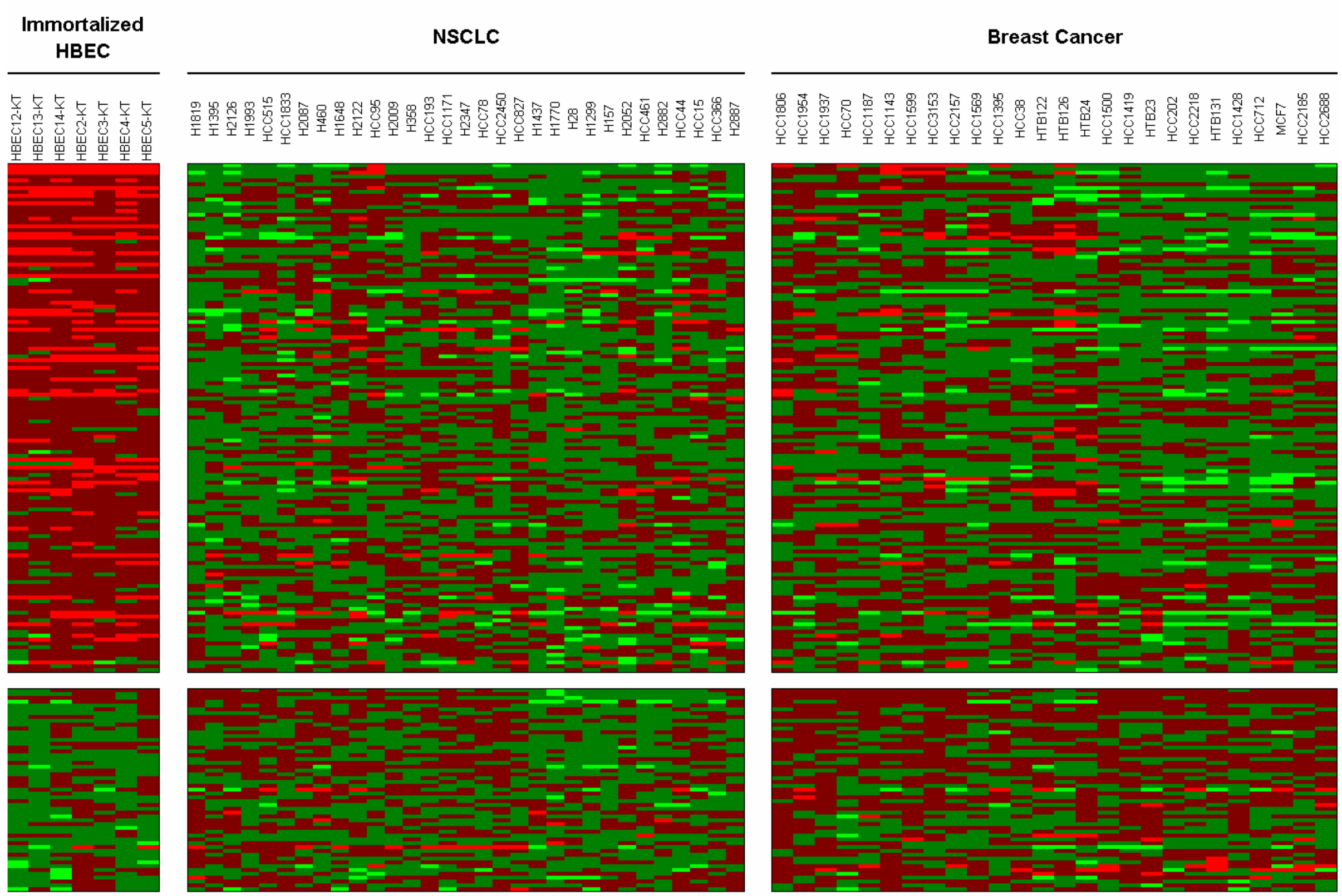
Immortalized HBEC
NSCLC
Breast Cancer
HBEC12-KT HBEC13-KT HBEC14-KT HBEC2-KT HBEC3-KT HBEC4-KT HBEC5-KT
H1819 H1395 H2126 H1993 HCC515 HCC1833 H2087 H460 H1648 H2122 HCC95 H2009 H358 HCC193 HCC1171 H2347 HCC78 HCC2450 HCC827 H1437 H1770 H28 H1299 H157 H2052 HCC461 H2882 HCC44 HCC15 HCC366 H2887
HCC1806 HCC1954 HCC1937 HCC70 HCC1187 HCC1143 HCC1599 HCC3153 HCC2157 HCC1569 HCC1395 HCC38 HTB122 HTB126 HTB24 HCC1500 HCC1419 HTB23 HCC202 HCC2218 HTB131 HCC1428 HCC712 MCF7 HCC2185 HCC2688

B

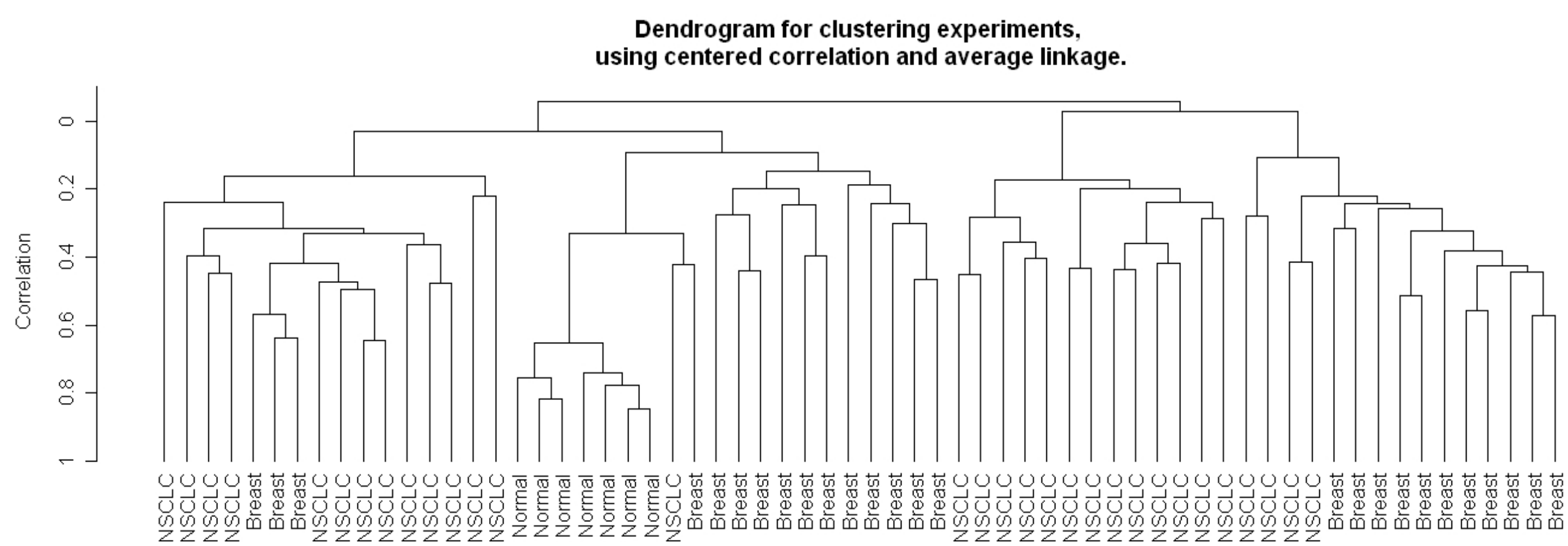
Dendrogram for clustering experiments,
using centered correlation and average linkage.
Correlation
0 0.2 0.4 0.6 0.8 1
NSCLC NSCLC NSCLC NSCLC Breast Breast Breast NSCLC NSCLC NSCLC NSCLC NSCLC NSCLC NSCLC NSCLC NSCLC Normal Normal Normal Normal Normal Normal Normal NSCLC Breast Breast Breast Breast Breast Breast Breast Breast Breast Breast Breast Breast NSCLC NSCLC NSCLC NSCLC NSCLC NSCLC NSCLC NSCLC NSCLC NSCLC NSCLC NSCLC NSCLC NSCLC NSCLC NSCLC NSCLC Breast Breast Breast Breast Breast Breast Breast Breast Breast Breast Breast
