# Supplementary material for: A Genome-Wide Screen for Promoter Methylation in Lung Cancer Identifies Novel Methylation Markers for Multiple Malignancies
Source: PLoS Med. 2006 Dec 26;3(12):e486. doi: 10.1371/journal.pmed.0030486 (PMC1716188; doi:10.1371/journal.pmed.0030486)
Supplement: Supplementary file 7 [file pmed.0030486.sg007.pdf]

Fig. S7

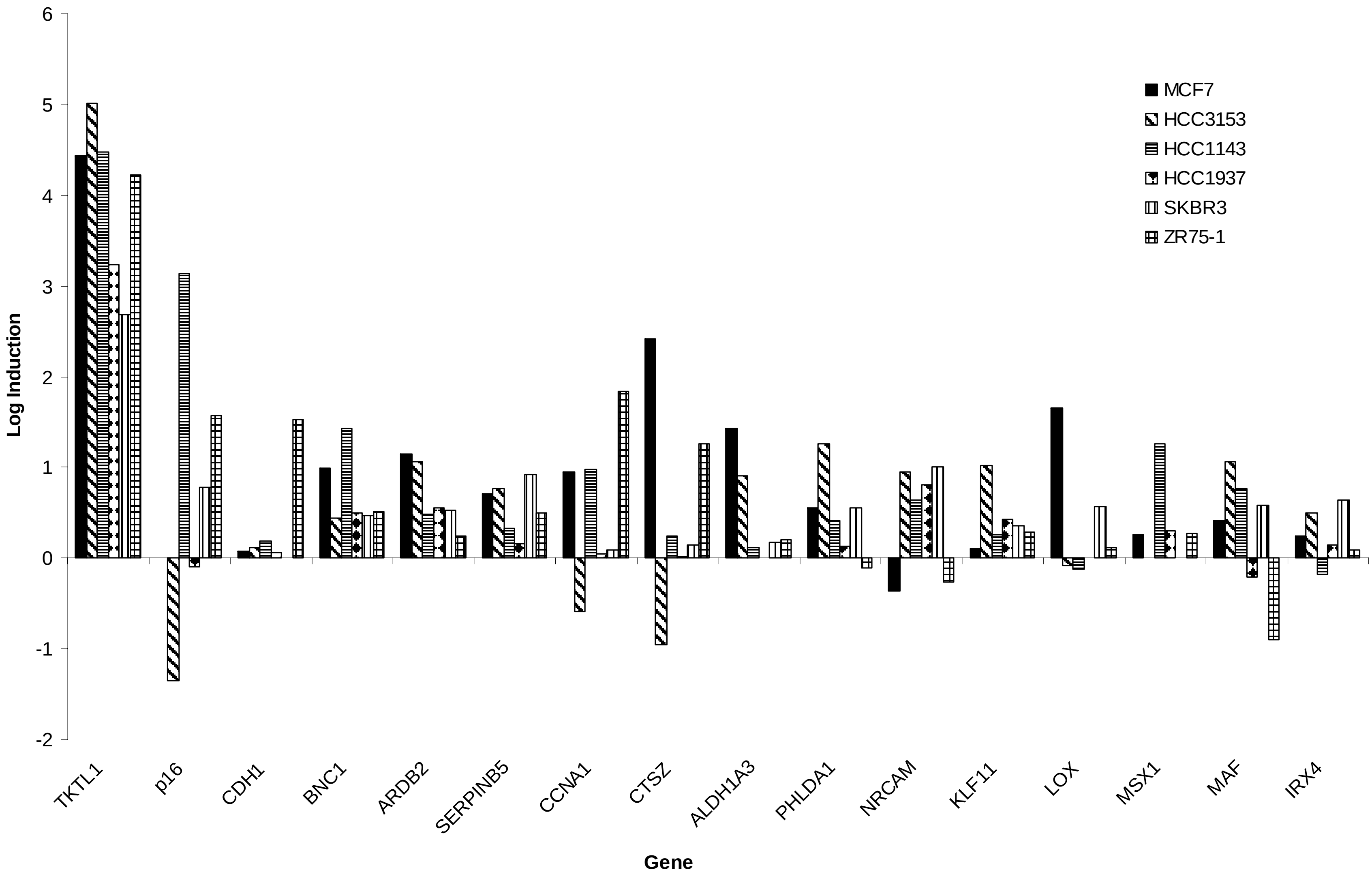

6
5
4
3
2
1
0
-1
-2
Log Induction
MCF7
HCC3153
HCC1143
HCC1937
SKBR3
ZR75-1
TKTL1
p16
CDH1
BNC1
ARDB2
SERPINB5
CCNA1
CTSZ
ALDH1A3
PHLDA1
NRCAM
KLF11
LOX
MSX1
MAF
IRX4
Gene
