# Supplementary material for: A Genome-Wide Screen for Promoter Methylation in Lung Cancer Identifies Novel Methylation Markers for Multiple Malignancies
Source: PLoS Med. 2006 Dec 26;3(12):e486. doi: 10.1371/journal.pmed.0030486 (PMC1716188; doi:10.1371/journal.pmed.0030486)
Supplement: Supplementary file 11 [file pmed.0030486.sg011.pdf]

Fig. S11

Methylation Analysis of *LOX*

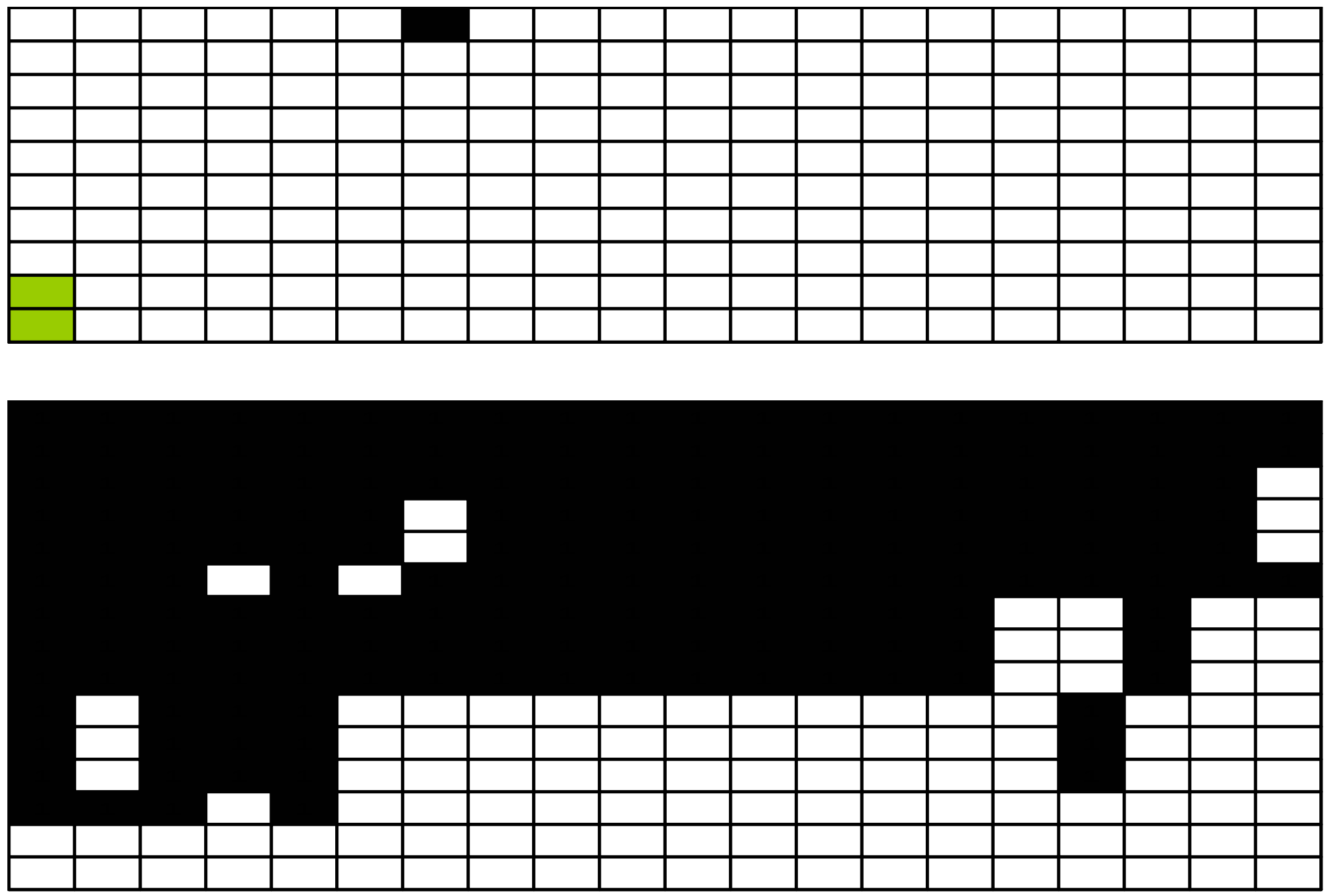

■ Methylated
□ Unmethylated
■ No data
