# Supplementary material for: A Genome-Wide Screen for Promoter Methylation in Lung Cancer Identifies Novel Methylation Markers for Multiple Malignancies
Source: PLoS Med. 2006 Dec 26;3(12):e486. doi: 10.1371/journal.pmed.0030486 (PMC1716188; doi:10.1371/journal.pmed.0030486)

Fig. S12

Methylation Analysis of *CTSZ*

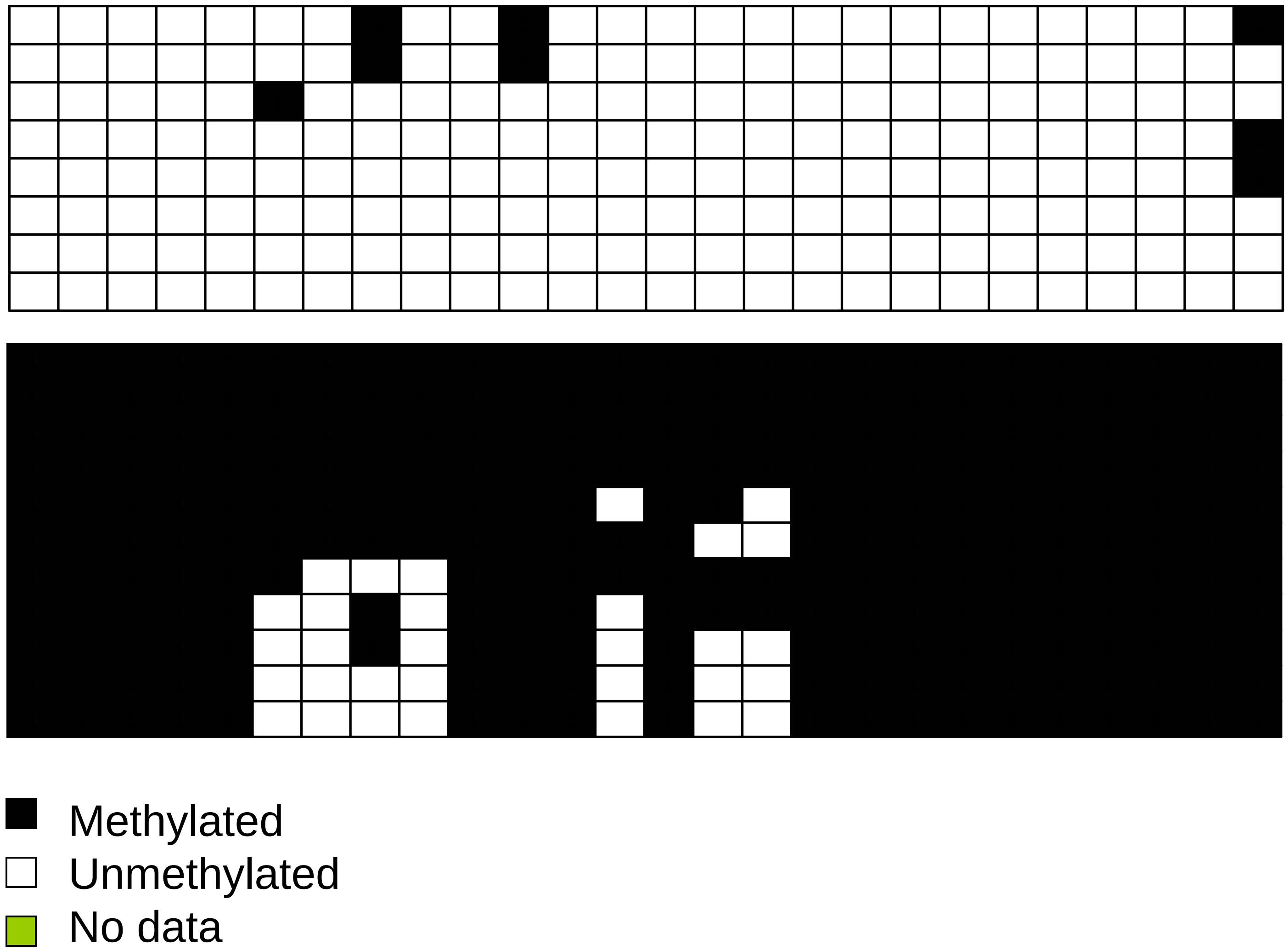

Supplement: Supplementary file 12 [file pmed.0030486.sg012.pdf]
