# Supplementary material for: A Genome-Wide Screen for Promoter Methylation in Lung Cancer Identifies Novel Methylation Markers for Multiple Malignancies
Source: PLoS Med. 2006 Dec 26;3(12):e486. doi: 10.1371/journal.pmed.0030486 (PMC1716188; doi:10.1371/journal.pmed.0030486)

Fig. S14

Methylation Analysis of *CCNA1*

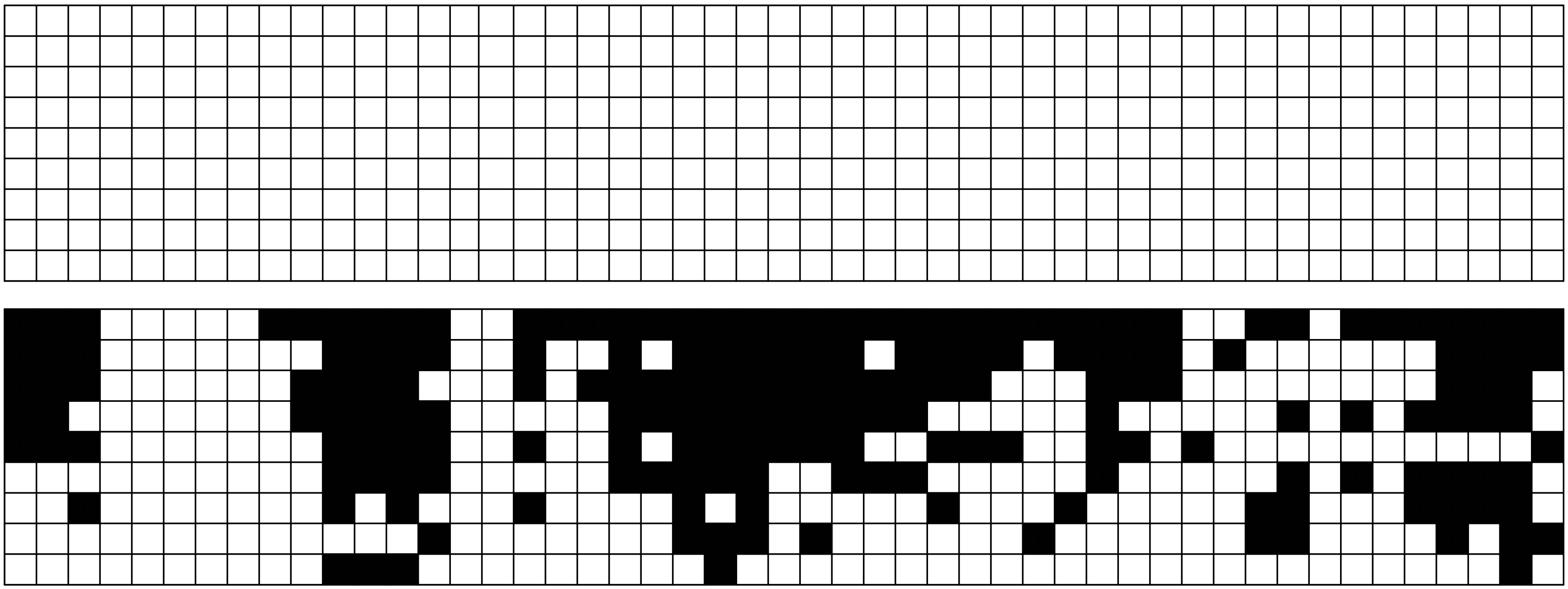

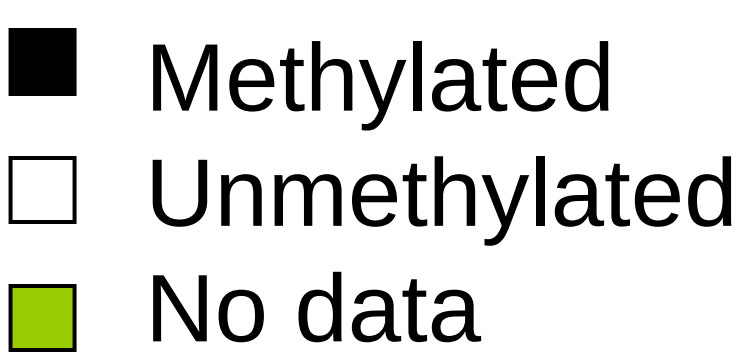

Supplement: Supplementary file 14 [file pmed.0030486.sg014.pdf]
